# Spatial and Temporal Variation in Fine Particulate Matter Mass and Chemical Composition: The Middle East Consortium for Aerosol Research Study

**DOI:** 10.1155/2014/878704

**Published:** 2014-06-18

**Authors:** Ziad Abdeen, Radwan Qasrawi, Jongbae Heo, Bo Wu, Jacob Shpund, Arye Vanger, Geula Sharf, Tamar Moise, Shmuel Brenner, Khaled Nassar, Rami Saleh, Qusai M. Al-Mahasneh, Jeremy A. Sarnat, James J. Schauer

**Affiliations:** ^1^Al Quads University (AQU), Beit Hanina, P.O. Box 51000, Jerusalem, Palestine; ^2^Environmental Chemistry and Technology Program, University of Wisconsin-Madison, Madison, WI 53706, USA; ^3^Institute of Applied Ecology, Chinese Academy of Sciences, Shenyang 110016, China; ^4^Israel Union for Environment Defense (IUED), 65134 Tel Aviv, Israel; ^5^Arava Institute for Environmental Studies (AIES), 88840 Hevel Eilot, Israel; ^6^Jordan Society for Sustainable Development (JSSD), Amman 840251, Jordan; ^7^Department of Environmental Health, Emory University, Atlanta, GA 30322, USA

## Abstract

Ambient fine particulate matter (PM_2.5_) samples were collected from January to December 2007 to investigate the sources and chemical speciation in Palestine, Jordan, and Israel. The 24-h PM_2.5 _samples were collected on 6-day intervals at eleven urban and rural sites simultaneously. Major chemical components including metals, ions, and organic and elemental carbon were analyzed. The mass concentrations of PM_2.5 _across the 11 sites varied from 20.6 to 40.3 *μ*g/m^3^, with an average of 28.7 *μ*g/m^3^. Seasonal variation of PM_2.5 _concentrations was substantial, with higher average concentrations (37.3 *μ*g/m^3^) in the summer (April–June) months compared to winter (October–December) months (26.0 *μ*g/m^3^) due mainly to high contributions of sulfate and crustal components. PM_2.5_ concentrations in the spring were greatly impacted by regional dust storms. Carbonaceous mass was the most abundant component, contributing 40% to the total PM_2.5 _mass averaged across the eleven sites. Crustal components averaged 19.1% of the PM_2.5 _mass and sulfate, ammonium, and nitrate accounted for 16.2%, 6.4%, and 3.7%, respectively, of the total PM_2.5_ mass. The results of this study demonstrate the need to better protect the health and welfare of the residents on both sides of the Jordan River in the Middle East.

## 1. Introduction

Atmospheric particulate matter (PM) is a complex, heterogeneous mixture, whose physical size distribution and chemical composition change in time and space, and is dependent on various emissions sources, atmospheric chemistry, and meteorological conditions [1, 2]. Atmospheric PM has important health and environmental impacts including (a) long-range transport of toxic materials; (b) visibility degradation; (c) hydrologic cycle combined with global climate change; and (d) radiation balance of the Earth [1–3]. There have been hundreds of epidemiologic and toxicologic studies that have shown associations between PM, specifically emitted by major mobile and stationary combustion sources, and excess morbidity and mortality due mainly to respiratory and cardiovascular disease [4–6].

The vast majority of these studies examining air pollution exposures and health have been conducted in the US and Western Europe, where standards for emissions and ambient air quality have been largely successful in reducing average outdoor pollution levels [7, 8]. Regulation of air pollution in the Middle East region is, at best, in its initial stages, with air quality standards for gaseous pollutants typically higher than those measured in Europe and North America [9–15].

Previous limited monitoring studies throughout the Middle East have indicated that PM mass concentrations are elevated throughout this region [14, 16, 17]. Information about PM composition, a likely factor associated with its toxicity, is virtually nonexistent. To our knowledge, no studies have been conducted, regionally, throughout the Middle East examining both levels and spatiotemporal trends of fine particulate matter (PM_2.5_) components. A greater understanding of PM_2.5_ composition, from a broader regional perspective, is of substantial interest for several reasons. First, because ongoing research is providing evidence that different components of PM_2.5_ have different risk factors [18], effective control strategies aimed at protecting public health need to be directed at those components that pose the greatest risk. Second, this information provides the foundation to develop effective control strategies, since the various components are related to different emission sources. Third, air quality regulations have largely focused on local or national control strategies but it is likely that emissions from countries that share the same airshed, such as Palestine, Israel, and Jordan, impact each other and that regional multinational air quality control strategies will need to be developed to best protect human health.

This current analysis provides results on PM_2.5_ concentrations and chemical composition collected as part of a novel monitoring study conducted over a relatively large geographical area within the Middle East (Palestine, Jordan, and Israel). The Middle East Consortium for Aerosol Research Study (MECARS) examines PM levels and seasonal variation for a range of chemical components, including water-soluble ions, carbonaceous species, and inorganic elements, and compares ambient concentrations of these components among adjacent sites which are separated by political boundaries (e.g., Eilat-Israel and Aqaba-Jordan) in an effort to better understand the sources of these chemical components. Although a number of studies have examined the spatiotemporal evaluation and characteristics of PM_2.5_ chemical components and sources within an airshed, there are still uncertainties in addressing the spatial variability. Therefore, the results from this study can provide better understanding of the spatial differences of sources and chemical components of PM_2.5_ in different geographic regions and also be used to make more effective regulatory policies.

## 2. Measurement

### 2.1. Sampling Sites

**Figure 1:**
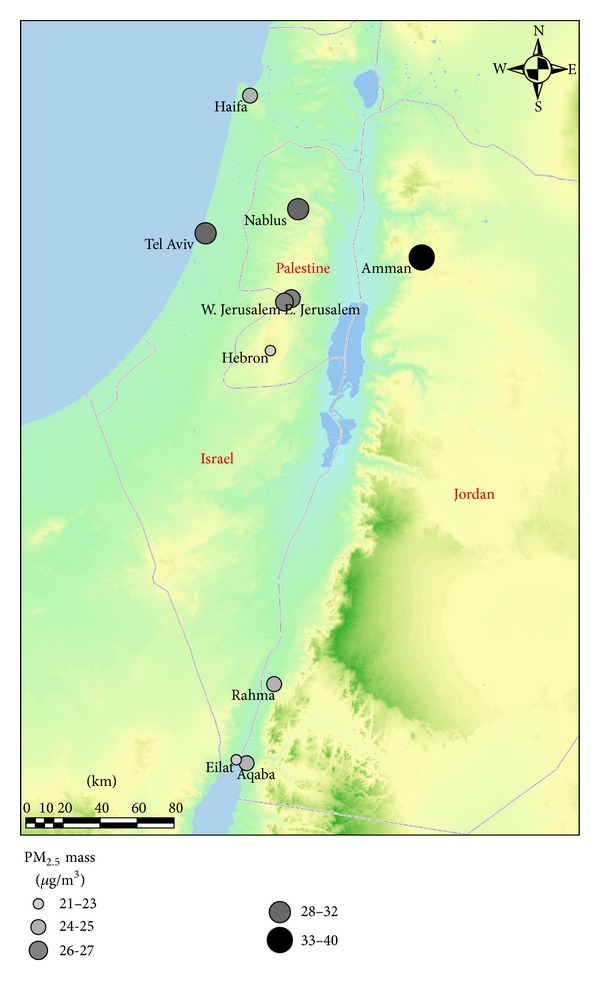
Sample site locations and their annual average PM_2.5_ mass concentrations.

PM samples were collected at eleven ambient air quality monitoring stations in Palestine (Nablus, East Jerusalem, and Hebron), Jordan (Amman, Aqaba, Rahma, and Zarka), and Israel (West Jerusalem, Eilat, Tel Aviv, and Haifa). The locations of the eleven stations are shown in Figure 1. Collectively, these stations were established as part of MECARS in 2007. These ambient monitoring stations were situated in populated areas, with the exception of the Rahma desert site and were intended to provide information pertaining to population exposure to PM. Special emphasis was placed on a comparison among different monitoring sites located in a single airshed. For example, West Jerusalem and East Jerusalem are part of the same urban area, and while Nablus and Hebron are only located ~50 km from each other, different in lifestyle, economic activity, vehicle fleet composition, and socioeconomic indicators may influence the outdoor PM_2.5_ concentrations and composition in each location. The two adjacent cities of Aqaba and Eilat differ greatly in size, economic character, building conditions, and lifestyle and are effectively isolated from each other at ground level by the international border. Concurrent monitoring of ambient PM_2.5_ was expected to elucidate differences among exposures in adjacent cities and reveal the causes of these differences. The sampling sites and associated economic and social differences across the sites have been more detailed in Sarnat et al. [19].

All of the monitoring sites were selected to minimize potential impacts from localized sources and activities. Sites were located in large clearings on relatively high ground and were situated a reasonable distance from busy thoroughfares and pollutant industrial point sources. The PM sampling stations used by the MECARS were situated on the roofs of buildings, resulting in an effective inlet height between 14 and 20 m above the ground. Therefore, the sampling stations were representative of urban background levels of PM concentrations for each site [20].

### 2.2. Sample Collection and Analysis

PM_2.5_ samples were collected every 6th day during 2007 (62 sampling events at each site), effectively once per week on alternating days of the week, to provide weekday and weekend measurements. Multichannel air samplers, designed and built specifically for this project by URG Corp (Chapel Hill, NC, USA), were comprised of two PM_2.5_ sampling trains, each of which operated at a total flow rate of 16.7 liters per minute (LPM). Each sampling train consisted of a sample inlet, a PM_2.5_ cyclone, a flow splitter, and two 8.35 LPM sample legs. All components of the sampler that were exposed to the aerosol sample flow were fabricated from Teflon-coated aluminum or Teflon-coated stainless steel. One sample train contained two filter holders used to collect particulate matter on 47 mm Teflon membrane filters for mass determination and analysis of inorganic particulate matter components. The second sampler leg contained one 47 mm quartz fiber filter (QFF) that collected particulate matter used for analysis of organic carbon (OC) and elemental carbon (EC) as well as individual organic compounds. The 4th sampling leg was replaced with a spacer to maintain standard flow rates and standard sampler construction and was not used for sampling. Air sampling flow rates on the sampler were controlled with critical orifices and the samplers were started at midnight on each sampling day and shut off after 24 hours using electronically controlled timers. Flow rates were measured with calibrated rotameters before and after sampling. Monthly field and lab blanks were analyzed for quality control and quality assurance and to blank corrections. The sampling system can be found in the supporting information (see Figure S1 in Supplementary Material available online at http://dx.doi.org/10.1155/2014/878704) and additional information on the collecting system has been previously published in von Schneidemesser et al. [21] and Sarnat et al. [19].

Prior to sample collection the QFFs were baked at 550°C for a minimum of 16 hours to remove residual carbonaceous material. Before and after sample collection, the QFFs were stored in prebaked aluminum foil in plastic Petri dishes sealed with Teflon tape. Each sample collected on the quartz fiber filter was analyzed for elemental and organic carbon (ECOC) by thermal-optical analysis (Sunset Laboratory, Inc., Forest Grove, OR) using the ACE-Asia method [22].

The Teflon filters were used to collect and measure PM_2.5_ mass, gravimetrically. Water-soluble ions and major and trace elements concentrations were determined by analyzing the collected PM_2.5_ mass using ion chromatography [23] and by X-ray fluorescence (XRF), respectively. The XRF analysis was conducted at the Desert Research Institute (DRI) in Reno, Nevada, using the EPA Compendium Method 10-3.3 [24]. The samples were stored frozen at all times after sampling and before chemical analysis except during shipping where they were kept cold to avoid volatility losses and chemical transformation. Explicit details of the filter samples handling and storage have been found in previously published works [25, 26]. QA/QC was performed to trace any contamination due to sample handling, with 12 sets of field blanks (one per sampling month) for each site. The blank filters were handled in the same manners as other 24-hour samples. All measurements were blank subtracted using the results of analyses of the blank filters. The field blank values for PM_2.5_ mass were not statistically different from zero. A mean of blank values for other chemical constituents varied, and the results can be found in Table S1. Meteorological data (temperature, air pressure, humidity, solar radiation, wind speed, and wind direction) were collected from locally equipped monitoring stations. In the Middle East areas, the weather changes from season to season, showing that from May to October is summer with hot and dry weather, from December to January is the coldest weather with high precipitation, and spring is short and lasts a little less than a month around April. For the present paper, calendar quarters (Q1 = January–March; Q2 = April–June; Q3 = July–September; Q4 = October–December) were used to estimate monthly trends in PM_2.5_ mass and chemical compounds.

## 3. Result and Discussion

### 3.1. PM_2.5_ Mass Concentrations

The average PM_2.5_ mass concentrations across the 11 sites ranged between 20.6 and 40.3 *μ*g/m^3^, with an overall average of 28.7 *μ*g/m^3^. These differences were expected based on the diverse site locations (Figure 1) and different local pollution sources. In these areas the key primary PM sources are energy production, industrial activities, and vehicular emissions, as well as dust [27].

Data analysis for Q4 and Q2 months indicated higher total PM_2.5_ concentrations during the warmer months of the year. In the Q2, an average across-site concentration of 37.3 *μ*g/m^3^ was measured, with a range of 12.2 *μ*g/m^3^ to 133 *μ*g/m^3^. The Q4 values averaged 20.2 *μ*g/m^3^, with a range of 5.7 *μ*g/m^3^ to 66.6 *μ*g/m^3^. The relative high concentrations in the warmer months were mainly driven by the synoptic wind conditions (i.e., the Red Sea Trough and Saharan Cyclone), which lead to elevated dust concentrations that occur in April-May in the region [19]. Among the sites, annual concentrations were highest in Amman, with an average concentration of 40.3 *μ*g/m^3^ and a maximum concentration of 162.0 *μ*g/m^3^. This high annual concentration is likely attributable to local anthropogenic emissions (industrial emissions and heavy vehicular traffic) of particulate matter compared to the other sites. During this study period, lead gasoline was used in Jordan, but not used in Israel, and traffic volumes were higher than other sites in Jordan. This could differentiate Amman from the other sites. While mass concentrations measured in this study were substantially lower than PM_2.5_ concentrations measured in many Asian cities [28–33], the measurements taken in Jordan, Palestine, and Israel showed that each of the eleven sampling sites had average annual PM_2.5_ concentrations above the US Environmental Protection Agency standard (15 *μ*g/m^3^) and World Health Organization guidelines (10 *μ*g/m^3^). A couple of sites (i.e., Haifa, Eilat, and Rahma) had annual averages below the new European Union standard (25 *μ*g/m^3^) which is, in general, comparable to PM_2.5_ concentrations measured at urban background sites in various European and American cities, where average levels are 20 to 30 *μ*g/m^3^ and 6 to 31.3 *μ*g/m^3^, respectively [34, 35].

### 3.2. Dust

The dust concentration contributing to PM_2.5_ mass across the sites was estimated by the common metal oxides of crustal elements (i.e., SiO_2_, Al_2_O_3_, K_2_O, CaO, Fe_2_O_3_, TiO_2,_ and MnO_2_) [36] at the sites as shown in the following:

(1) Dust=2.139×Si+1.899×Al+1.205×K+1.4×Ca +1.43×Fe+1.668×Ti+1.582×Mn.

**Figure 2:**
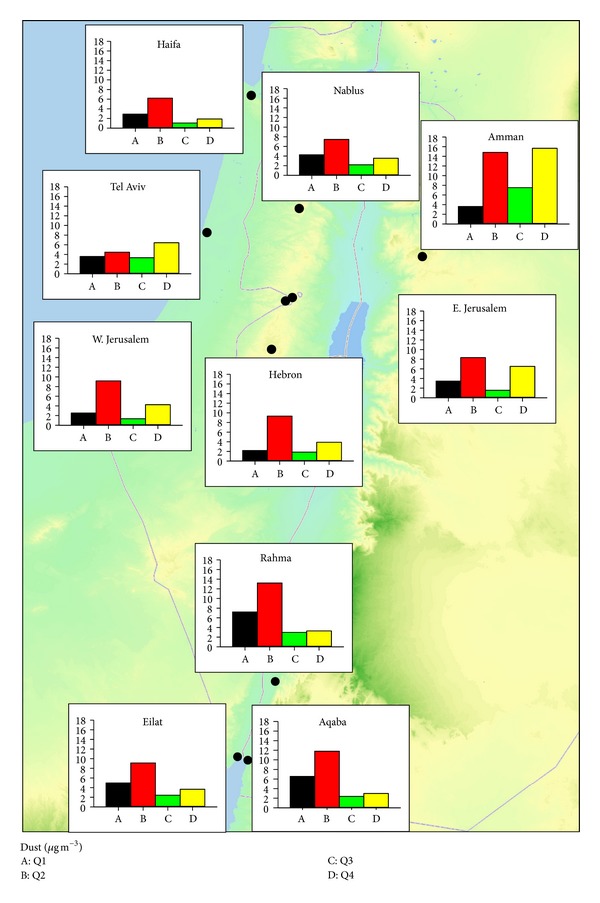
Seasonal trends of dust concentrations across sites.

Results from the dust calculation show that dust components contribute to 19% of total PM_2.5_ mass over the study period in the region, and high dust contributions to PM_2.5_ are observed at four of the eleven sites, ranging from 24% to 27% in Eilat, Aqaba, Amman, and Rahma (Table 1). Dust contributions for the other sites are less than the average annual contribution among all sites, ranging from 13% to 18% in Haifa and Zarqa, respectively. This level is moderately higher than 5 to 10% contributions to PM_2.5_ mass concentrations at 60 rural, urban, and curbside sites across Europe as well as East Asian Countries [37, 38] but is not a dominant component of the PM_2.5_. This is an important result of the current study, because the expectation that much of the PM_2.5_ is dust undermines the need to address particulate matter air pollution in this study region. In addition, PM_2.5_ mass levels at monitoring stations in Israel are impacted much less than the PM_10_ (less than 10 *μ*m aerodynamic diameter) and is elevated by a factor of 2 while PM_10_ can go high by a factor more than 10 to reach even 1500 and 2000 *μ*g/m^3^ (data not shown). Thus, the difference in dust contribution to PM_2.5_ between sites can be explained.  Figure 2 shows spatial and seasonal trends in dust concentrations across the sites during the sampling year. The dust concentrations exhibit a distinct seasonal variation with high enhancement in the Q2 months. Two extreme dust events on May 30th and October 9th coincided with the sampling period and resulted in elevated PM_2.5_ mass concentrations, with mean PM_2.5_ mass concentrations of 99.7 *μ*g/m^3^ and 54.9 *μ*g/m^3^, respectively, among all sites. Consequently, the dust components of PM_2.5_ were elevated across all sites during these dust events, with cross-site average of 50.3 *μ*g/m^3^ and 14.0 *μ*g/m^3^ for May and October, respectively. Overall, due to dust storm events between April and May, there is a significant enhancement of dust concentrations during the Q2 months in the region.

**Figure 3:**
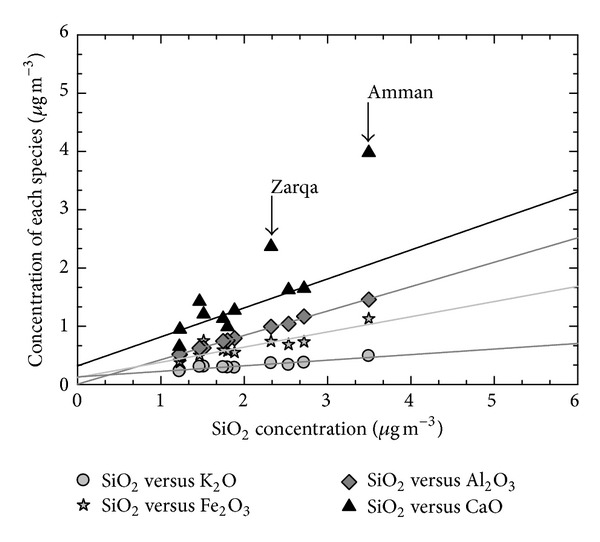
Comparison of dust oxides between sampling sites (both Amman and Zarqa data points were excluded from the CaO and SiO_2_ regression).

Urban dusts (i.e., crustal PM components) are typically associated with site-specific conditions, such as the type and abundance of industrial activities, nature of emissions, traffic load, traffic flow, and more [39]. Elemental ratios of dust components can be used to estimate types and/or sources of soil/dust particles [40]. In this study, mean SiO_2_ concentration was plotted against the mean concentration of four other crustal metal oxides for each site to evaluate the similarity of dust type in PM_2.5_ in the region (Figure 3). There are similar ratios and strong spatiotemporal correlations for K_2_O/SiO_2_, Fe_2_O_3_/SiO_2_, and Al_2_O_3_/SiO_2_ across the sites, implying that these dust components are relatively homogenous and no specific local sources are contributing to these components throughout the entire region. For the CaO/SiO_2_ ratio, Amman and Zarqa sites had higher values than other sites, which indicate that site-specific local emissions contribute to additional Ca mass at both sites. For this reason, we have excluded these two sites in the regression of CaO and SiO_2_ shown in Figure 3. In general, Ca/Si mass ratio estimated from chemical analyses for paved, unpaved road and soil has been about 0.3–0.6 [40, 41]. While Ca is the fifth most abundant element in earth metals, it is also largely emitted from anthropogenic activities including cement kilns and limestone quarries, as well as coal fire power plants [42]. It should be noted that there was a large construction in Amman during this study period [19], thus, the heavy use of construction-related gypsum (i.e., CaSO_4_·H_2_O) might be affecting high Ca concentrations in Amman relative to the other sites. The Zarqa site is located 15 km to the northeast of the Amman site and because of this geographic proximity, it is possible that the calcium-rich dust emitted from the construction activity in Amman may also have affected the Ca level in Zarqa [19]. In addition, there are numerous industrial processing sites in Zarqa, which may be contributing to additional Ca.

### 3.3. Inorganic Water Soluble Ions

Inorganic water soluble ions (i.e., SO_4_^2−^, NO_3_^−^, Cl^−^, NH_4_^+^, Na^+^, and K^+^) detected by ion chromatograph showed that annual mean concentrations for total ion matters varied by site. High ion levels were present at Amman, Zarqa, and Haifa, where a large number of local and heavy industrial activities exist relative to the other sites (Figure S2). The ionic mass fraction contributed approximately 28% of the total PM_2.5_ mass across all sites, with a range of 21% in Amman to 37% in Haifa. In particular, key ionic ions were SO_4_^2−^ and NH_4_^+^, explaining around 23% of total PM_2.5_ mass in the region (Table S2). The total ion contribution to the PM_2.5_ mass in this study appears to be lower than those from previous studies conducted at many urban and rural sites in Europe, North America, and Asia, where secondary sulfate, nitrate, and ammonium together (i.e., SO_4_^2−^, NO_3_^−^, and NH_4_^+^) account for 50% of the PM_2.5_ mass concentrations [38, 43–47]. This difference seems to be due primarily to the lower concentrations of NO_3_^−^, which is formed through a photochemical reaction of NO_*x*_.

In this study, SO_4_^2−^ and NH_4_^+^ showed significantly high (*r* > 0.83) correlations among the intercorrelation for each pair of ion species at almost every site, whereas the relationship between NO_3_^−^ and NH_4_^+^ was not statistically significant (Table S4). In addition, the ambient particulate concentration ratio of NH_4_^+^ to SO_4_^2−^ less than 1.5 indicates an ammonium poor ambient atmosphere, which limits the formation of ammonium nitrate concentrations [14]. In this study, the ratio of NH_4_^+^/SO_4_^2−^ shows around 0.4. Therefore, it could be explained that after ammonia was fully neutralized by formation of ammonium sulfate aerosols, not enough ammonia remained (NH_3_-limited environment) to form fine particles of ammonium nitrate in the region.

The mass ratio NO_3_^−^/SO_4_^2−^ has been used as an indicator of the relative prevalence of stationary and mobile sources of sulfur and nitrogen in the atmosphere of polluted areas [32, 48–50]. Wang et al. [51] report that the estimated ratios of NO_*x*_ to SO_*x*_ from the emissions of gasoline and diesel fuel burning are 13 : 1 and 8 : 1, respectively, and are 1 : 2 from coal burning through emission factors and demonstrate that it is reasonable to use SO_4_^2−^ as an indicator of stationary emission and NO_3_^−^ of mobile emission. Therefore, ratio values between 0.30 and 0.50 were ascribed to industrial use of high sulfur coal [49], while lower values (0.13 ± 0.06) were inferred as indicating predominance of stationary source emissions over traffic emissions [50]. The NO_3_^−^/SO_4_^2−^ ratio in this study ranged from 0.15 at Haifa to 0.28 at Tel Aviv. This suggests that the contributions of mobile emissions to fine particles at all sites are less significant than those of industrial origin. Overall, ammonium nitrate concentrations are not found to be important for PM_2.5_ in this study region.

Seasonal trends in SO_4_^2−^ and NO_3_^−^ concentrations for the 11 sites are presented in Figures S4–S6. As expected, the SO_4_^2−^ concentrations were highest between April and August, whereas the NO_3_^−^ exhibited lowest concentrations in these months. In general, the oxidation reaction of SO_2_ via OH radical is enhanced in summer due to strong solar radiation, resulting in high SO_4_^2−^ concentrations in summer [52]. The observed seasonal trends of these two ions over the study region are well in accord with documented evidence. Amman showed no strong seasonality in the SO_4_^2−^ concentrations due to local construction activities as explained before.

### 3.4. Ion Balance

**Figure 4:**
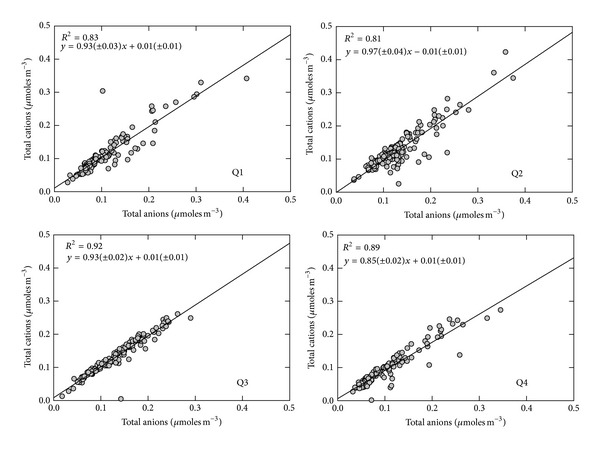
Correlations between a total of cations and anions over the study period.

The neutralization of the acidity present in aerosols is important to aerosol hygroscopicity, heterogeneous chemistry, and gas/particle partitioning. To better understand the degree of neutralization of aerosols, an ion balance can be investigated to compare cation and anion concentrations in the aerosol [44]. The aerosol composition in the sampled region shows a broad variability, with the mass concentrations of each individual species varying up to an order of magnitude throughout the sampling period. When total equivalents of cations (∑Na^+^ + K^+^ + NH_4_^+^) are plotted against total equivalents of anions (∑Cl^−^ + NO_3_^−^ + 2SO_4_^2−^) as shown in Figure 4, the slope of the regression lines for Q1, Q2, Q3, and Q4 was 0.93 (*r* ^2^ = 0.83), 0.97 (*r* ^2^ = 0.81), 0.93 (*r* ^2^ = 0.92), and 0.85 (*r* ^2^ = 0.89), respectively. All seasons except for the Q4 showed that the slope is not very different from 1.0, which indicates all cations considered are fully neutralized for sulfate and nitrate aerosols. For the Q4, the slope of the regression line is different from 1.0, weighted towards total anions which means anions are not completely neutralized by considered cations. This difference is most likely from reduced emissions of ammonia associated lower temperatures and differences in agricultural practices in the Q4 months.

### 3.5. Trace Elements

Trace elements including heavy metals are significant components of PM in urban environments. These elements are of particular concern due to their persistence in the environmental media and their human toxicity [53, 54]. In particular, nonbiodegradability of heavy metals leads to their accumulation in the environment [55].

A cluster analysis [56] is an exploratory data analysis tool that yields groups of elements that have common sources or common meteorological factors controlling their concentrations. Metals from samples with 90% of their concentrations below the detection limits were eliminated from the cluster analysis to increase analytical precision. 18 trace elements: Al, Si, P, S, Cl, K, Ca, Ti, V, Mn, Fe, Ni, Cu, Zn, Br, Sr, Yt, and Pb, were used in the analysis. Table S3 summarizes annual average concentrations of each metal across eleven sites. Results from the analysis found that there are three groups of a common source and one group of a site-specific source among the observed trace elements across the sites (Table 2).

Group 1 is characterized by high loading of Al, Si, Fe, Mn, Ca, K, and Sr, most of which are crustal elements, and appears to indicate dust aerosol impacts in the region. Group 2 is strongly correlated with sulfur and phosphate. This group represents industrial activities including coal fired power plants as well as regional loading of SO_4_^2−^ in this study area. Because phosphorus pentasulfide (P_2_S_5_) is commonly used for lubricating oil and grease additives and for organophosphorus materials [57], it is possible that this specific source contributes to the high correlation between P and S in this group. Group 3 is characterized by strong correlations between Pb, Zn, and Br. In general, the increased concentrations and/or the strong correlation between Pb, Zn, and Br are found to be in traffic source profiles, including tire wear, brake lining, catalyst deterioration, and fuel combustion emissions [58–60]. Therefore, this group may be associated with the effects of mobile emissions in the region. Group 4 is categorized by Ni and V, which are predominantly emitted from fuel oil combustion, and thus represents fuel oil use in the region. This group is only identified in East Jerusalem, Rahma, Zarqa, and Haifa, suggesting site-specific local emissions. The results of chemical components analysis revealed that toxic metals in PM_2.5_ varied significantly across sample sites, with highest concentrations in Haifa and Zarqa. Haifa had significantly higher concentrations of nickel and zinc compared to the other sites which is likely a result of the combustion of residual oils used by ships, the stationary combustion of fuel oil, or by industrial activity. Likewise, Zarqa had significantly higher concentrations of lead in the PM_2.5_ samples than any other site, including Aqaba and Amman, suggesting that the high level of lead in Zarqa is not attributable to leaded gasoline fuel, a type of fuel still used in Jordan during the study period. However, for Rahma, there is high loading of Ni and Br in the group 4. The Rahma sample site is located in a desert area, representing a relatively remote rural environment. This site received high concentrations of Ni and Br which may be associated with well-known industrial sources of Br near the Dead Sea and sea salt which contains traces of Br. This site-specific Br emission can impact the ambient particles in Rahma during regional transportation, but the correct source characterization could not be captured by this cluster analysis. Further research with other air quality receptor methods is needed to better understand the sources.

### 3.6. Carbonaceous Species

Carbonaceous materials in PM_2.5_ are important components in both urban and rural areas due to comprising from 10 to 70% of total PM_2.5_ mass [61, 62]. Carbonaceous aerosols are directly emitted from diverse sources and formed by photochemical reactions, thus understanding of them and their sources is very important.

The statistical presentation of the analytical results for carbonaceous species present in PM_2.5_ samples is reported in Table 1. Carbonaceous material was the dominant contributor (50–60%) to PM_2.5_ mass in this study. The highest levels of PM_2.5_ OC were observed in Zarqa, Nablus, and Amman (Figure S3). OC and EC concentrations are the largest contributors to PM_2.5_ in the region with the possible exceptions of Eilat, Aqaba, and Rahma. This regional OC and EC PM_2.5_ contribution is similar to Europe and North America. Average OC and EC concentrations were 5.30 ± 4.03 and 2.06 ± 8.93 *μ*g/m^3^, respectively with a 2.57 ± 2.08 OC/EC ratio. The OC and EC concentrations in Tel Aviv were 38% and 50% higher than in Haifa. Similar results were reported in Osaka, Japan [63], which showed an average of 5.2 and 3.4 *μ*g/m^3^ OC and EC, respectively, and an OC/EC ratio of 1.53.

For OC and EC, between-site correlations among the sites were weak, representing large heterogeneity among the sites due to site specific local emission sources. Previously published work has extensively addressed the spatiotemporal trends of both OC and EC of PM_2.5_ in the Middle East [19]. Generally, the measured amount of carbonaceous components tended to increase from summer to winter. EC is typically emitted from diesel engines and exhibits high intraurban spatial heterogeneity given its local, primary source contributions. The present findings showed moderate to strong correlations (*R* ^2^ > 0.65) in EC concentrations among the large urban sites of West Jerusalem, Tel Aviv, and Amman. Moreover, the correlations were strong despite absolute differences in PM_2.5_ EC concentrations measured in each of these cities. The high EC correlations in these urban centers may be due to synoptic conditions (i.e., the influence of stagnation episodes) and commuter activity patterns. Similarly, weaker EC correlations among the other sites may point to the impact of specific EC sources beyond those of traffic-related emissions.

OC concentrations were corrected by a factor of 1.8 in order to assess the particulate organic matter (OM). This correction factor utilized corresponds to mildly oxidized organic material in the urban environment [64, 65]. OM concentrations were approximately five times greater than observed EC concentrations and ranged from 3.94 *μ*g/m^3^ to 15.40 *μ*g/m^3^, resulting in an annual average of 9.54 *μ*g/m^3^. OM was the third most abundant component of PM_2.5_ across sampling sites. In comparison, the OM annual average was greater than the 3.0 *μ*g/m^3^ measurement taken in Helsinki, Finland [66]; but lower than those measured in Sihwa, Korea [31], Seoul, Korea [67], Kaohsiung, Taiwan [29], and Shanghai [33], where values ranged from 9.8 *μ*g/m^3^ to 15.4 *μ*g/m^3^. The relationship between OM and EC carbon can provide some indication of the origin of carbonaceous particles [68, 69]. The OM to EC correlation (*R* ^2^ = 0.80) was strong compared to other studies [28, 31, 67].

**Figure 5:**
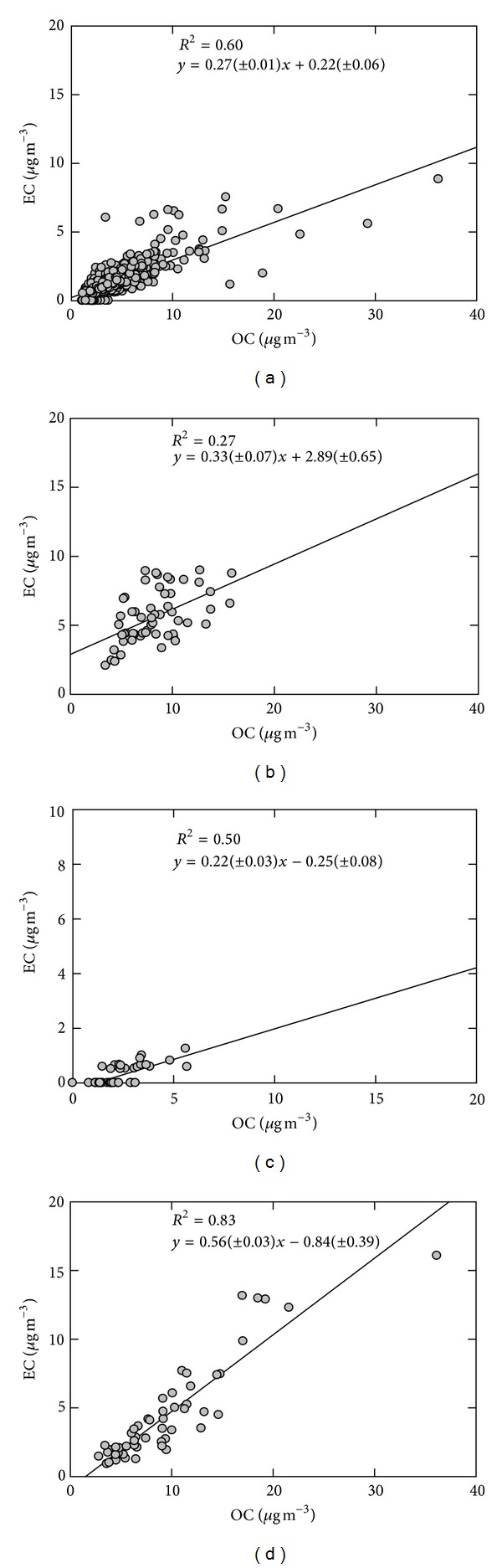
Comparison between OC and EC concentrations (graph (a) includes East Jerusalem, Hebron, Aqaba, Amman, Haifa, Eilat, Tel Aviv, and West Jerusalem; graphs (b, c, and d) indicate Nablus, Rachma, and Zarqa, resp.).

EC which is only emitted from combustion sources is often related to primary OC, and the relationship between two species can provide insight into their origins [62]. Previous studies have shown that OC and EC correlations at urban areas are higher than rural areas due to predominant urban activities, mostly motor vehicle emissions [70, 71]. Concentrations of EC are plotted against the corresponding OC concentrations for categorized sampling sites in Figure 5. Correlations are generally high (*r* > 0.5) at most sites where both EC and OC levels appear to be influenced by the same primary sources. However, there is lower correlation at Nablus site compared to the other sites due probably to a large amount of biogenic sources and potential gas-phase volatile organic compounds in Nablus. More details on the sources of OC and EC at these sites have been presented by von Schneidemesser et al. [21].

### 3.7. Secondary Organic Carbon (SOC) Estimation

Because OC can be derived from emitted particles as well as secondary organic aerosol, it was important to confirm the contributions of the primary and secondary organic carbon to carbonaceous aerosol for control of particulate pollution. Secondary organic carbon (SOC) is often a significant portion of OC in PM. Quantification of SOC is difficult because of the limited understanding of the molecular composition of SOC and the presence of a large and unknown number of individual secondary organic products. The concentration levels and seasonal patterns of SOC in the 11 sampling sites were examined by the EC tracer method [29, 66, 72]. In this method, SOC is calculated as follows:

(2) $$\begin{array}{c} \text{SOC}=\text{OC}-\left({\left[\frac{\text{OC}}{\text{EC}}\right]}_{\text{prim}}\times \text{EC}+{\text{OC}}_{\text{non-combustion}}\right). \end{array}$$

In this equation, EC is adopted as a “tracer” for calculating the abundance of primary OC based on EC being primary in origin and EC and OC having common emission sources [64, 69, 72]. A ratio of OC/EC that is characteristic of primary emissions, called (OC/EC)_prim_, hereafter, is used to estimate SOC. Ambient OC/EC exceeding the (OC/EC)_prim_ ratio is attributed to SOC. OC_non-combustion_ represents primary noncombustion emissions. A linear regression of a subset of data was applied in order to determine the value of (OC/EC)_prim_ and OC_non-combustion_. From the regression equation, (OC/EC)_prim_ was determined as the slope and OC_non-combustion_ as the* y*-intercept.

Due to the fact that photochemistry is important throughout the year in the Middle East, the minimum OC/EC ratio was estimated as the average of the lowest 5% of the OC/EC at each site and the lowest 10% of the OC/EC values at each site. Both approaches were used to evaluate the sensitivity of the calculation.

**Figure 6:**
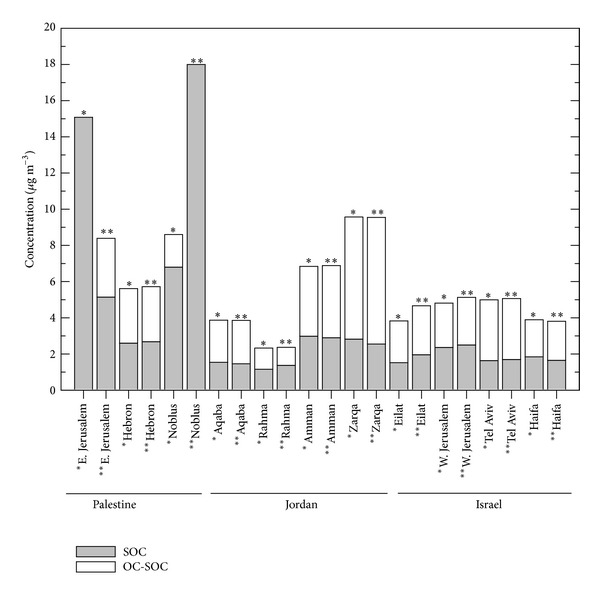
Calculated secondary organic aerosol during the study period (∗; lowest 5% of OC/EC ratio for Deming regression (*n* = 4 for each site), ∗∗; lowest 10% of OC/EC ratio for Deming regression (*n* = 6 for each site)).

As seen in Figure 6, the 5% and 10% assumption yielded virtually the same result for all sites except East Jerusalem and Nablus. The poor agreement between estimation approaches for these two sites suggests that the (OC/EC)_prim_ at these two sites varies over time due to different impacts of sources.

The average SOC level at all sites excluding East Jerusalem and Nablus for the entire sampling period was 2.71 *μ*gC/m^3^, contributing to 55.48% of the OC aerosol loadings. On a mass basis, the secondary organic aerosol (SOA) contributed 17.20% of the PM_2.5_ mass, when using a factor of 1.8 to convert SOC to SOA mass [64].

This estimation is in agreement with measurements in other studies of urban areas [64, 69, 72]. These results suggest that the formation of SOA due to the gas/particle conversion of gaseous hydrocarbon precursors is significant in urban locations and demonstrates the importance of identifying SOC precursors for effective reduction of aerosol loadings.

### 3.8. Mass Closure

Knowledge of the chemical composition of atmospheric aerosols and mass closure studies in the Middle East are needed for scientific and policy reasons. Aerosol chemical mass closure calculations were performed for the PM_2.5_ aerosol, for each of the 11 parallel samplings. The following eight aerosol components were used to calculate the PM_2.5_ mass closure which was then compared to the gravimetrically calculated PM_2.5_ mass: (1) OM, which was estimated by multiplying the OC by a factor of 1.8 [64]; (2) EC; (3) sulfate; (4) nitrate; (5) ammonium; (6) sea salt, estimated as [Cl] + 1.4486[Na], where 1.4486 is the ratio of the concentration of all elements except Cl to the Na concentration in sea salt [73]; (7) dust, estimated as 1.899[Al] + 2.138[Si] + 1.400[Ca] + 1.205[K] + 1.668[Ti] + 1.430[Fe] + 1.582[Mn]; and (8) other elements, which are the sum of the mass of all noncrustal/nonsea-salt elements measured by PIXE (S and K were excluded from this sum). Sulfate, nitrate, ammonium, and sea salt were obtained from the IC data of the PM_2.5_ samples.

**Figure 7:**
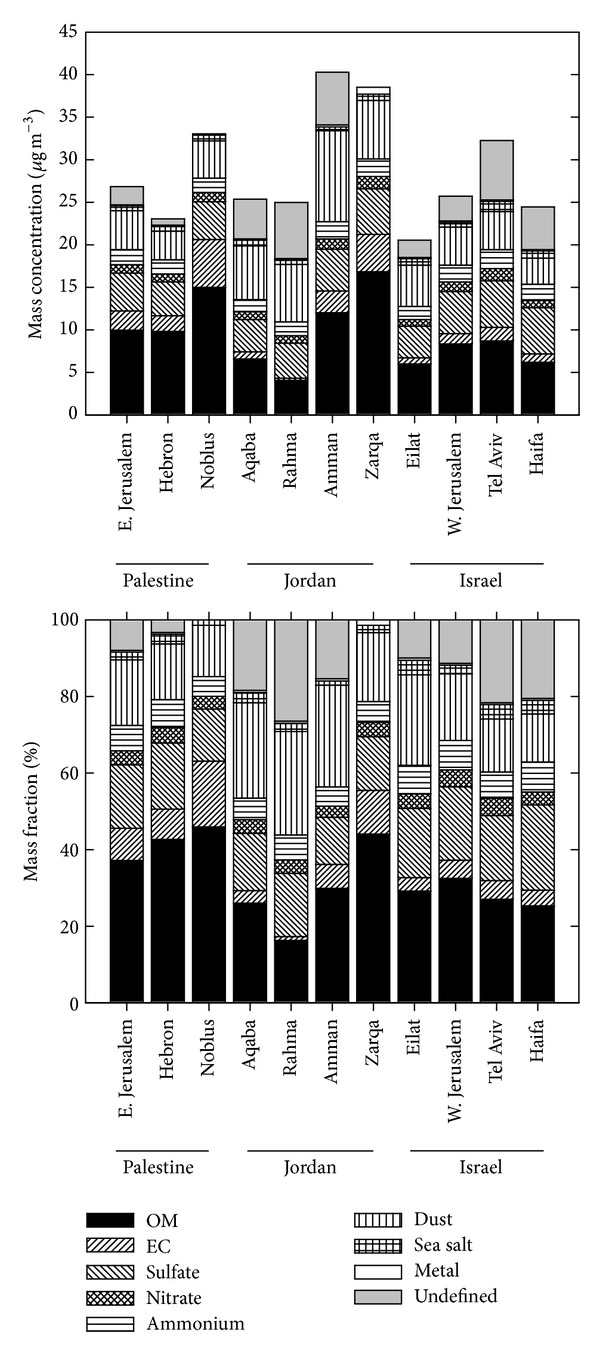
Annual average mass concentration (top) and mass fraction (bottom) of major PM_2.5_ chemical components for each site.

The relative contribution of identified chemical classes to the PM_2.5_ mass at each site is shown in Figure 7. The average concentrations of the eight aerosol components among all sites were as follows: OM = 9.54 *μ*g/m^3^, EC = 2.07 *μ*g/m^3^, ammonium = 1.83 *μ*g/m^3^, nitrate = 1.06 *μ*g/m^3^, sulfate = 4.65 *μ*g/m^3^, sea salt = 0.71 *μ*g/m^3^, dust = 5.47 *μ*g/m^3^, and other elements = 0.20 *μ*g/m^3^. The average total PM_2.5_ mass based on these 8 components was 25.53 *μ*g/m^3^ while the average gravimetric PM_2.5_ mass for all sites was 28.67 *μ*g/m^3^. Approximately 3.14 *μ*g/m^3^ or 10.96% of the PM_2.5_ mass was not accounted by this calculation. The percentage attributions of the gravimetric PM_2.5_ mass to each of the eight components types are given in Figure 7.

Relative contributions reflect differences in emission sources and processes controlling the aerosol composition [74]. The PM_2.5_ profiles varied among the eleven sites. Mineral components dominated the PM_2.5_ profile at Aqaba, Amman, Rachma, Tel Aviv, and West Jerusalem contributing 22.8%, 24.6%, 24.8%, 12.7%, and 15.87% to the total PM_2.5_ mass, respectively. The contribution of carbonaceous material to total mass ranged from 16.7% to 64.3% at the eleven sites and dominated the PM_2.5_ profile in Zarka and Nablus. The differences are likely to be due to variable contributions of local combustion sources such as residential heating with solid fuels (coal, wood), vehicle exhaust, biogenic emissions, and photochemical reactions [75]. The inorganic ion fraction constituted 20.3% to 35.2% of the total PM_2.5_ mass and was the main contributor in Haifa and Eliat. Trace elements contributed only 0.83% to the PM_2.5_ at the eleven sites. The contribution of sea salt to the PM_2.5_ mass across the sites varied from 3.8% in Tel Aviv and Haifa, close to the coast, to 1.1% in Amman, land inwards. This result indicates the variability and the gradient of sea spray emissions to PM_2.5_ from coastal to inland areas in the region.

Similar conclusions representing observed PM_2.5_ mass closure problems due to unidentified chemical components are drawn by other studies [75, 76]. Several factors may be responsible for the discrepancies observed in mass closure. Estimates of the organic matter amounts and the crustal material components were simplified and contain high uncertainties. Organic matter estimates may have been probably underestimated by using 1.8 as the conversion factor at all study sites. Values as high as 2.1 have been widely used in the literature as the conversion factor [77, 78]. Large uncertainties are also associated with the contribution of crustal components. Estimation of minerals and trace elements included only the oxides, however, metals in dust may be in other forms [76, 79]. Furthermore, unidentified water content in the PM_2.5_ samples may lead to the PM_2.5_ mass closure problems. Other possible causes for PM_2.5_ mass closure problem could be artifacts during sampling and systematic errors in chemical analysis.

## 4. Conclusions

For the first time ambient PM_2.5_ mass concentrations were monitored in three countries in the Middle East (Jordan, Palestinian Authority, and Israel) for 52 consecutive weeks. Mass concentrations, chemical compositions, seasonal variation, and site-to-site variations of the PM_2.5_ data were examined. The mass concentrations of PM_2.5_ across the 11 sites varied from 20.1 to 40.3 *μ*g/m^3^, with an average of 28.5 *μ*g/m^3^. PM_2.5_ mass concentrations exhibited strong monthly variation across 11 sites, with the maximum difference occurring in May and November. Average OC and EC concentrations were 5.30 ± 4.03 and 2.06 ± 8.93 *μ*g/m^3^, respectively, and the average OC ratio was 2.57 ± 2.08. The study also revealed that heavy metals in PM_2.5_ varied significantly across the sites. Sulfate and nitrate are by far the most prominent anions and ammonium is the prominent cation in the PM_2.5_ fraction at all sites.

Obvious seasonal variations of PM_2.5_ mass concentrations were observed. PM_2.5_ concentrations were highest during the Q2 and tended to be at their lowest concentrations during the Q4. In particular, ambient PM_2.5_ levels in April-May were clearly impacted by dust storms.

Overall, carbonaceous aerosol was the most abundant species and averaged 40% of the PM_2.5_ mass, while crustal matter and ionic species, namely, sulfate, nitrate, and ammonium, were also major components. Measured species accounted for 87% of the observed mass. Additional work is needed to improve the mass balance and to obtain the source profiles needed to use this data for source apportionment.

## Supplementary Material

We provide field blank values of each chemical species, showing quality control and quality assurance for this study, and detailed the observed concentrations of ionic species and metals across 11 sampling sites, as well as the sampling equipment used to collect ambient PM_2.5_ samples. In addition, inter-correlation results among each pair of ionic species for each site and spatial trends in concentrations of ionic species across the sampling sites are provided.

## Figures and Tables

**Table 1:** Annual average concentrations of PM_2.5_ components across the eleven sampling sites, MECARS-2007 (unit: microgram per cubic meter).

Site	Statistics	PM_2.5_ mass	OC	EC	SO_4_^2−^	NO_3_^−^	NH_4_^+^	Dust	Toxic metal	Metal
All sites										
	Minimum	5.6	N.D	N.D	N.D	N.D	0.0	N.D	N.D	N.D
	Mean	28.7	5.3	2.1	4.6	1.1	1.8	5.5	0.1	0.5
	Std. deviation	17.8	4.0	2.2	2.6	0.9	1.0	8.6	0.4	0.5
	Maximum	162.0	36.2	16.1	14.9	8.1	6.8	75.4	4.8	3.3

Palestine										
E. Jerusalem	Minimum	9.7	2.0	0.9	0.9	0.2	0.3	0.4	N.D	0.1
	Mean	26.8	5.5	2.3	4.5	1.0	1.8	4.6	0.1	0.4
	Std. deviation	17.1	4.3	1.4	2.4	0.7	0.9	8.1	0.1	0.4
	Maximum	117.9	29.3	6.6	10.7	3.6	4.1	53.7	0.4	2.8
Hebron	Minimum	8.1	2.0	0.7	1.1	0.2	0.4	0.3	N.D	0.1
	Mean	23.1	5.5	1.8	4.0	0.9	1.7	3.4	0.1	0.3
	Std. deviation	10.8	2.7	0.9	2.3	0.6	0.9	4.0	0.1	0.2
	Maximum	65.0	13.3	5.1	13.0	3.2	4.3	20.7	0.4	0.9
Nablus	Minimum	13.3	3.5	2.1	1.0	0.4	0.3	0.3	0.1	0.1
	Mean	30.9	8.3	5.6	4.4	1.1	1.7	4.4	0.1	0.4
	Std. deviation	12.4	2.9	1.9	2.5	0.7	0.9	6.5	0.1	0.3
	Maximum	85.1	15.9	9.0	11.0	3.7	4.0	39.0	0.4	2.0

Jordan										
Aqaba	Minimum	10.3	1.1	N.D	0.8	0.2	0.0	N.D	N.D	N.D
	Mean	25.4	3.7	0.8	3.8	0.9	1.4	6.3	0.1	0.4
	Std. deviation	16.5	1.6	0.6	1.6	0.5	0.8	9.5	0.1	0.4
	Maximum	114.2	8.9	3.0	7.7	3.4	4.3	58.5	0.2	2.5
Rahma	Minimum	8.3	N.D	N.D	0.9	0.2	0.3	0.4	N.D	0.1
	Mean	25.0	2.3	0.3	4.1	0.9	1.6	6.7	0.1	0.5
	Std. deviation	18.7	1.1	0.3	2.1	0.6	0.9	11.2	0.1	0.5
	Maximum	132.0	5.7	1.3	11.5	3.0	4.3	72.0	0.2	3.1
Amman	Minimum	12.4	1.8	0.6	1.6	0.3	0.6	0.3	0.1	0.1
	Mean	40.3	6.7	2.5	4.9	1.2	2.0	10.7	0.1	0.7
	Std. deviation	25.4	4.0	1.7	2.3	0.7	1.1	13.7	0.1	0.5
	Maximum	162.0	20.4	7.5	11.5	3.9	5.8	75.4	0.5	3.0
Zarqa	Minimum	12.6	2.8	0.9	1.3	0.1	0.5	0.5	0.1	0.1
	Mean	37.5	9.4	4.4	5.4	1.4	2.1	6.9	0.7	0.6
	Std. deviation	19.4	5.8	3.6	2.5	1.3	1.1	9.3	1.1	0.5
	Maximum	113.0	36.2	16.1	11.8	7.6	5.9	58.9	4.8	2.7

Israel										
Eilat	Minimum	5.6	1.2	N.D	N.D	N.D	0.4	0.6	0.1	0.1
	Mean	20.6	3.3	0.7	3.7	0.8	1.5	4.9	0.1	0.4
	Std. deviation	9.6	1.5	0.5	1.8	0.8	0.9	5.2	0.1	0.3
	Maximum	53.7	7.1	1.6	7.2	4.8	4.9	23.6	0.2	1.3
W. Jerusalem	Minimum	10.3	1.4	N.D	0.9	0.2	0.3	0.2	N.D	0.1
	Mean	25.7	4.6	1.2	4.9	1.1	2.0	4.5	0.1	0.4
	Std. deviation	18.2	4.9	1.2	2.8	0.7	1.0	7.7	0.1	0.5
	Maximum	116.6	36.2	8.8	14.2	3.0	4.7	50.2	0.6	2.9
Tel Aviv	Minimum	13.0	1.6	N.D	1.2	N.D	0.4	0.4	0.1	0.2
	Mean	32.2	4.8	1.6	5.5	1.4	2.2	4.5	0.1	0.8
	Std. deviation	12.5	2.6	0.9	3.3	1.6	1.3	3.9	0.1	0.7
	Maximum	80.7	12.7	3.7	14.9	8.1	6.8	17.5	0.2	3.3
Haifa	Minimum	7.8	1.1	N.D	0.7	N.D	0.0	0.2	N.D	0.1
	Mean	24.4	3.4	1.0	5.4	0.8	1.9	3.1	0.1	0.4
	Std. deviation	17.0	2.9	0.7	3.3	0.6	1.1	6.8	0.1	0.4
	Maximum	106.8	22.6	4.8	14.9	3.1	5.6	48.5	0.4	2.8

N.D represents not detected.

**Table 2:** Cluster analysis results for metals observed at each site.

Site		Group 1	Group 2	Group 3	Group 4
Palestine	E. Jerusalem	Al, Ti, Si, Fe, Mn, Ca, K, Sr	P, S	Pb, Br, Zn	V, Ni
	Hebron	Al, Si, Fe, Ti, Mn, K, Ca, Sr	P, S		
	Nablus	Al, Si, Ti, Fe, Mn, K, Ca, Sr	P, S	Pb, Cu, Br,Yt	

Jordan	Aqaba	Al, Si, Fe, Ti, Ca, Mn, K, Sr		Pb, Zn	
	Rahma	Al, Si, Ti, Fe, Ca, Mn, K, Sr	P, S	Pb, Zn	Ni, Br
	Amman	Al, Si, Fe, Ti, Mn, K, Ca, Sr, V	P, S	Pb, Br	
	Zarqa	Al, Si, Ti, Fe, K, Sr, Ca	P, S	Pb, Yt, Cl, Br	V, Ni

Israel	Eilat	Al, Si, Ti, Fe, Mn, Ca, K, Sr	P, S	Pb, Zn	
	W. Jerusalem	Al, Si, Ti, Fe, Mn, Ca, K, Sr	P, S	Pb, Br, Zn	
	Tel Aviv	Al, Si, Ti, Fe, Mn, K, Sr	P, S	Pb, Zn	
	Haifa	Al, Si, Ti, Fe, Mn, Ca, K, Sr	P, S	Pb, Br	V, Ni
