# Supplementary material for: Spatial and Temporal Variation in Fine Particulate Matter Mass and Chemical Composition: The Middle East Consortium for Aerosol Research Study
Source: ScientificWorldJournal. 2014 Jun 18;2014:878704. doi: 10.1155/2014/878704 (PMC4090475; doi:10.1155/2014/878704)
Supplement: Supplementary file 1 — We provide field blank values of each chemical species, showing quality control and quality assurance for this study, and detailed the observed concentrations of ionic species and metals across 11 sampling sites, as well as the sampling equipment used to collect ambient PM2.5 samples. In addition, inter-correlation results among each pair of ionic species for each site and spatial trends in concentrations of ionic species across the sampling sites are provided. [file 878704.f1.pdf]

## Supplemental Materials

Table S1. Summary of field blank values (μg/m$^3$) for each of chemical compounds.

| Chemical species | Field blanks (μg/m$^3$) |
| --- | --- |
| OC | 0.4837 (± 0.0244) |
| EC | 0.0090 (± 0.0022) |
| $SO_4^{2-}$ | 1.7088 (± 0.2049) |
| $NO_3^-$ | 1.8571 (± 0.0496) |
| $Cl^-$ | 0.7626 (± 0.0228) |
| $NH_4^+$ | 0.1145 (± 0.0378) |
| $Na^+$ | 0.0000 (± 0.0315) |
| $K^+$ | 0.0000 (± 0.0166) |
| Na | 0.0226 (± 0.0070) |
| Mg | 0.0096 (± 0.0017) |
| Al | 0.0049 (± 0.0013) |
| Si | 0.0065 (± 0.0029) |
| P | 0.0019 (± 0.0005) |
| S | 0.0118 (± 0.0095) |
| Cl | 0.0051 (± 0.0046) |
| K | 0.0017 (± 0.0010) |
| Ca | 0.0082 (± 0.0041) |
| Ti | 0.0004 (± 0.0001) |
| V | 0.0001 (± 0.0000) |
| Cr | 0.0004 (± 0.0001) |
| Mn | 0.0008 (± 0.0002) |
| Fe | 0.0024 (± 0.0013) |
| Co | 0.0000 (± 0.0000) |
| Ni | 0.0005 (± 0.0001) |
| Cu | 0.0005 (± 0.0002) |
| Zn | 0.0014 (± 0.0004) |
| Br | 0.0004 (± 0.0002) |
| Sr | 0.0005 (± 0.0001) |
| Pb | 0.0012 (± 0.0002) |

Table S2. Annual average concentrations of inorganic water soluble ions across eleven sites (unit: microgram per cubic meter).

| Site | | $PM_{2.5}$ mass | $SO_4^{2-}$ | $NO_3^-$ | $Cl^-$ | $NH_4^+$ | $Na^+$ | $K^+$ |
|---|---|---|---|---|---|---|---|---|
| All Sites | Min | 5.64 | N.D | N.D | N.D | 0.01 | N.D | N.D |
| | Mean | 28.66 | 4.65 | 1.06 | 0.23 | 1.83 | 0.33 | 0.09 |
| | Std | 17.75 | 2.58 | 0.87 | 0.29 | 1.03 | 0.29 | 0.09 |
| | Max | 161.97 | 14.93 | 8.14 | 2.78 | 6.79 | 3.09 | 1.22 |
| E. Jerusalem | Min | 9.72 | 0.88 | 0.18 | N.D | 0.33 | N.D | N.D |
| | Mean | 26.84 | 4.45 | 0.98 | 0.20 | 1.78 | 0.25 | 0.08 |
| | Std | 17.10 | 2.42 | 0.67 | 0.16 | 0.93 | 0.22 | 0.10 |
| | Max | 117.92 | 10.71 | 3.58 | 0.60 | 4.07 | 1.00 | 0.68 |
| Hebron | Min | 8.10 | 1.09 | 0.22 | 0.04 | 0.37 | N.D | N.D |
| | Mean | 23.06 | 3.98 | 0.93 | 0.22 | 1.67 | 0.26 | 0.08 |
| | Std | 10.85 | 2.35 | 0.55 | 0.17 | 0.90 | 0.22 | 0.07 |
| | Max | 64.99 | 12.99 | 3.20 | 0.78 | 4.26 | 0.86 | 0.32 |
| Nablus | Min | 13.29 | 0.98 | 0.39 | 0.01 | 0.32 | 0.01 | 0.02 |
| | Mean | 30.89 | 4.44 | 1.08 | 0.25 | 1.69 | 0.30 | 0.11 |
| | Std | 12.43 | 2.49 | 0.67 | 0.20 | 0.92 | 0.22 | 0.06 |
| | Max | 85.13 | 10.99 | 3.73 | 0.78 | 3.98 | 0.99 | 0.31 |
| Aqaba | Min | 10.33 | 0.77 | 0.15 | N.D | 0.01 | N.D | N.D |
| | Mean | 25.37 | 3.81 | 0.90 | 0.17 | 1.42 | 0.35 | 0.08 |
| | Std | 16.48 | 1.57 | 0.53 | 0.23 | 0.75 | 0.25 | 0.05 |
| | Max | 114.16 | 7.69 | 3.38 | 1.27 | 4.28 | 1.06 | 0.25 |
| Rahma | Min | 8.34 | 0.93 | 0.23 | 0.02 | 0.31 | 0.01 | 0.02 |
| | Mean | 24.98 | 4.13 | 0.87 | 0.14 | 1.63 | 0.29 | 0.11 |
| | Std | 18.73 | 2.07 | 0.58 | 0.15 | 0.89 | 0.19 | 0.06 |
| | Max | 131.97 | 11.48 | 2.95 | 0.61 | 4.31 | 0.83 | 0.27 |
| Amman | Min | 12.37 | 1.64 | 0.33 | N.D | 0.62 | N.D | N.D |
| | Mean | 40.29 | 4.93 | 1.19 | 0.15 | 2.03 | 0.20 | 0.09 |
| | Std | 25.44 | 2.31 | 0.73 | 0.17 | 1.05 | 0.17 | 0.10 |
| | Max | 161.97 | 11.52 | 3.88 | 0.83 | 5.82 | 0.75 | 0.68 |
| Zarqa | Min | 12.63 | 1.33 | 0.08 | 0.01 | 0.47 | N.D | N.D |
| | Mean | 37.52 | 5.37 | 1.44 | 0.32 | 2.06 | 0.29 | 0.11 |
| | Std | 19.42 | 2.52 | 1.25 | 0.32 | 1.13 | 0.22 | 0.08 |
| | Max | 113.01 | 11.83 | 7.55 | 1.77 | 5.92 | 0.78 | 0.30 |
| Eilat | Min | 5.64 | N.D | N.D | 0.01 | 0.40 | 0.02 | 0.02 |
| | Mean | 20.56 | 3.72 | 0.77 | 0.30 | 1.54 | 0.34 | 0.11 |
| | Std | 9.60 | 1.78 | 0.79 | 0.49 | 0.85 | 0.22 | 0.06 |
| | Max | 53.65 | 7.22 | 4.81 | 2.78 | 4.88 | 0.97 | 0.31 |
| W. | Min | 10.29 | 0.85 | 0.18 | 0.01 | 0.34 | 0.01 | N.D |

| | | | | | | | | |
|---|---|---|---|---|---|---|---|---|
| Jerusalem | Mean | 25.72 | 4.93 | 1.14 | 0.19 | 1.97 | 0.27 | 0.08 |
| | Std | 18.19 | 2.83 | 0.68 | 0.21 | 1.03 | 0.24 | 0.18 |
| | Max | 116.62 | 14.15 | 2.99 | 1.05 | 4.69 | 1.13 | 1.22 |
| | Min | 13.04 | 1.24 | N.D | N.D | 0.38 | 0.02 | N.D |
| Tel Aviv | Mean | 32.25 | 5.48 | 1.44 | 0.38 | 2.22 | 0.58 | 0.09 |
| | Std | 12.47 | 3.25 | 1.56 | 0.53 | 1.32 | 0.54 | 0.06 |
| | Max | 80.65 | 14.90 | 8.14 | 2.39 | 6.79 | 3.09 | 0.31 |
| | Min | 7.84 | 0.73 | N.D | N.D | 0.03 | N.D | N.D |
| Haifa | Mean | 24.44 | 5.44 | 0.83 | 0.26 | 1.91 | 0.42 | 0.09 |
| | Std | 16.98 | 3.28 | 0.64 | 0.26 | 1.15 | 0.28 | 0.09 |
| | Max | 106.81 | 14.93 | 3.09 | 0.95 | 5.60 | 1.39 | 0.59 |

*not detected, min=minimum, Std=Standard deviation, Max=Maximum

Table S3. Annual average concentrations of each trace metal across eleven sites (unit: microgram per cubic meter).

| Site | Statistics | Al | Si | P | S | Cl | K | Ca | Ti | V | Mn | Fe | Ni | Cu | Zn | Br | Sr | Pb |
|---|---|---|---|---|---|---|---|---|---|---|---|---|---|---|---|---|---|---|
| All sites | Min | N.D* | N.D | N.D | N.D | N.D | N.D | N.D | N.D | N.D | N.D | N.D | N.D | N.D | N.D | N.D | N.D | N.D |
| | Mean | 0.445 | 0.932 | 0.071 | 1.770 | 0.178 | 0.267 | 1.131 | 0.041 | 0.005 | 0.007 | 0.450 | 0.001 | 0.004 | 0.031 | 0.012 | 0.003 | 0.072 |
| | Std | 0.782 | 1.634 | 0.045 | 0.954 | 0.299 | 0.284 | 1.649 | 0.071 | 0.005 | 0.013 | 0.700 | 0.003 | 0.006 | 0.053 | 0.023 | 0.007 | 0.359 |
| | Max | 6.431 | 13.707 | 0.346 | 5.720 | 2.585 | 2.360 | 15.284 | 0.590 | 0.043 | 0.120 | 6.534 | 0.052 | 0.056 | 0.516 | 0.266 | 0.060 | 4.748 |
| E. Jerusalem | Min | N.D | N.D | N.D | 0.266 | 0.008 | 0.040 | 0.136 | N.D | N.D | N.D | 0.054 | N.D | N.D | N.D | N.D | N.D | N.D |
| | Mean | 0.394 | 0.816 | 0.067 | 1.766 | 0.117 | 0.247 | 0.808 | 0.037 | 0.004 | 0.005 | 0.414 | 0.001 | 0.004 | 0.024 | 0.005 | 0.002 | 0.010 |
| | Std | 0.758 | 1.551 | 0.037 | 0.905 | 0.162 | 0.316 | 1.367 | 0.072 | 0.003 | 0.012 | 0.704 | 0.001 | 0.009 | 0.037 | 0.006 | 0.006 | 0.017 |
| | Max | 4.857 | 9.852 | 0.150 | 3.973 | 0.726 | 2.020 | 9.577 | 0.470 | 0.015 | 0.077 | 4.672 | 0.005 | 0.056 | 0.270 | 0.025 | 0.038 | 0.072 |
| Hebron | Min | N.D | N.D | N.D | 0.173 | 0.006 | 0.045 | 0.085 | N.D | N.D | N.D | 0.022 | N.D | N.D | N.D | N.D | N.D | N.D |
| | Mean | 0.272 | 0.574 | 0.062 | 1.571 | 0.088 | 0.200 | 0.675 | 0.025 | 0.003 | 0.004 | 0.264 | 0.000 | 0.003 | 0.025 | 0.005 | 0.001 | 0.008 |
| | Std | 0.386 | 0.831 | 0.039 | 0.905 | 0.108 | 0.144 | 0.552 | 0.035 | 0.003 | 0.007 | 0.350 | 0.001 | 0.003 | 0.058 | 0.007 | 0.003 | 0.011 |
| | Max | 1.943 | 4.148 | 0.168 | 4.400 | 0.546 | 0.802 | 3.098 | 0.181 | 0.014 | 0.032 | 1.750 | 0.006 | 0.013 | 0.356 | 0.032 | 0.015 | 0.042 |
| Nablus | Min | N.D | N.D | N.D | 0.273 | 0.010 | 0.078 | 0.060 | N.D | N.D | N.D | 0.052 | N.D | N.D | 0.011 | N.D | N.D | N.D |
| | Mean | 0.330 | 0.684 | 0.074 | 1.791 | 0.135 | 0.253 | 1.017 | 0.031 | 0.005 | 0.004 | 0.352 | 0.001 | 0.003 | 0.044 | 0.005 | 0.002 | 0.009 |
| | Std | 0.643 | 1.317 | 0.042 | 0.960 | 0.160 | 0.218 | 0.948 | 0.058 | 0.003 | 0.010 | 0.564 | 0.001 | 0.004 | 0.063 | 0.005 | 0.005 | 0.016 |
| | Max | 3.653 | 7.462 | 0.181 | 4.306 | 0.576 | 1.491 | 6.517 | 0.334 | 0.014 | 0.050 | 3.200 | 0.007 | 0.017 | 0.423 | 0.021 | 0.023 | 0.085 |
| Aqaba | Min | N.D | N.D | N.D | N.D | N.D | N.D | N.D | N.D | N.D | N.D | N.D | N.D | N.D | N.D | N.D | N.D | N.D |
| | Mean | 0.552 | 1.183 | 0.080 | 1.481 | 0.080 | 0.278 | 1.159 | 0.051 | 0.004 | 0.007 | 0.480 | 0.000 | 0.001 | 0.016 | 0.013 | 0.005 | 0.019 |
| | Std | 0.848 | 1.789 | 0.065 | 0.755 | 0.180 | 0.288 | 1.808 | 0.074 | 0.004 | 0.014 | 0.735 | 0.001 | 0.002 | 0.011 | 0.014 | 0.009 | 0.027 |
| | Max | 5.080 | 10.847 | 0.346 | 3.781 | 0.584 | 1.944 | 11.455 | 0.446 | 0.017 | 0.083 | 4.474 | 0.005 | 0.010 | 0.042 | 0.049 | 0.045 | 0.154 |
| Rahma | Min | 0.037 | N.D | N.D | 0.253 | N.D | 0.074 | 0.110 | N.D | 0.001 | N.D | 0.034 | N.D | N.D | N.D | N.D | N.D | N.D |
| | Mean | 0.618 | 1.270 | 0.075 | 1.641 | 0.117 | 0.311 | 1.176 | 0.053 | 0.005 | 0.007 | 0.511 | 0.001 | 0.001 | 0.019 | 0.013 | 0.005 | 0.013 |
| | Std | 1.043 | 2.118 | 0.041 | 0.792 | 0.181 | 0.342 | 2.096 | 0.091 | 0.004 | 0.015 | 0.866 | 0.001 | 0.002 | 0.015 | 0.012 | 0.010 | 0.025 |
| | Max | 6.431 | 13.138 | 0.168 | 3.973 | 0.671 | 2.360 | 14.173 | 0.570 | 0.020 | 0.097 | 5.539 | 0.005 | 0.009 | 0.078 | 0.047 | 0.060 | 0.157 |
| Amman | Min | 0.030 | N.D | N.D | 0.575 | 0.015 | 0.050 | 0.075 | N.D | N.D | N.D | 0.020 | N.D | N.D | 0.004 | 0.005 | N.D | N.D |
| | Mean | 0.772 | 1.633 | 0.073 | 1.933 | 0.220 | 0.405 | 2.842 | 0.075 | 0.005 | 0.013 | 0.790 | 0.001 | 0.005 | 0.026 | 0.033 | 0.007 | 0.085 |
| | Std | 1.189 | 2.593 | 0.039 | 0.878 | 0.256 | 0.418 | 2.756 | 0.113 | 0.004 | 0.022 | 1.149 | 0.002 | 0.006 | 0.018 | 0.045 | 0.011 | 0.125 |
| | Max | 5.972 | 13.707 | 0.193 | 4.386 | 1.025 | 2.359 | 15.284 | 0.590 | 0.024 | 0.120 | 6.534 | 0.013 | 0.033 | 0.091 | 0.266 | 0.058 | 0.488 |
| Zarqa | Min | 0.037 | 0.028 | N.D | 0.363 | 0.009 | 0.057 | 0.177 | N.D | N.D | N.D | 0.034 | N.D | N.D | 0.004 | N.D | N.D | N.D |

| | | | | | | | | | | | | | | | | | | |
|---|---|---|---|---|---|---|---|---|---|---|---|---|---|---|---|---|---|---|
| | Mean | 0.525 | 1.085 | 0.068 | 1.838 | 0.227 | 0.303 | 1.690 | 0.047 | 0.006 | 0.012 | 0.517 | 0.001 | 0.005 | 0.086 | 0.031 | 0.003 | 0.593 |
| | Std | 0.879 | 1.814 | 0.043 | 0.945 | 0.302 | 0.294 | 1.715 | 0.076 | 0.005 | 0.015 | 0.747 | 0.002 | 0.007 | 0.112 | 0.040 | 0.007 | 1.046 |
| | Max | 5.376 | 10.960 | 0.183 | 4.695 | 1.830 | 2.046 | 10.996 | 0.464 | 0.016 | 0.074 | 4.563 | 0.007 | 0.025 | 0.516 | 0.255 | 0.042 | 4.748 |
| | Min | N.D | 0.053 | N.D | 0.334 | 0.007 | 0.060 | 0.069 | N.D | N.D | N.D | 0.056 | N.D | N.D | N.D | N.D | N.D | N.D |
| Eilat | Mean | 0.419 | 0.880 | 0.063 | 1.549 | 0.153 | 0.238 | 0.908 | 0.038 | 0.005 | 0.006 | 0.386 | 0.002 | 0.002 | 0.020 | 0.010 | 0.003 | 0.012 |
| | Std | 0.494 | 1.023 | 0.034 | 0.742 | 0.190 | 0.147 | 0.887 | 0.044 | 0.003 | 0.008 | 0.422 | 0.002 | 0.003 | 0.016 | 0.011 | 0.006 | 0.026 |
| | Max | 2.074 | 4.476 | 0.141 | 3.809 | 0.678 | 0.641 | 4.354 | 0.206 | 0.015 | 0.033 | 2.006 | 0.006 | 0.013 | 0.084 | 0.042 | 0.025 | 0.145 |
| | Min | N.D | N.D | N.D | 0.238 | N.D | 0.046 | 0.083 | N.D | N.D | N.D | 0.034 | N.D | N.D | N.D | N.D | N.D | N.D |
| W. Jerusalem | Mean | 0.400 | 0.841 | 0.069 | 1.830 | 0.117 | 0.243 | 0.706 | 0.037 | 0.004 | 0.005 | 0.402 | 0.001 | 0.003 | 0.024 | 0.004 | 0.002 | 0.010 |
| | Std | 0.747 | 1.522 | 0.039 | 0.952 | 0.197 | 0.331 | 1.207 | 0.067 | 0.003 | 0.011 | 0.655 | 0.001 | 0.004 | 0.053 | 0.007 | 0.006 | 0.019 |
| | Max | 4.741 | 9.540 | 0.176 | 4.680 | 0.922 | 1.853 | 8.440 | 0.424 | 0.014 | 0.061 | 4.155 | 0.005 | 0.018 | 0.403 | 0.042 | 0.034 | 0.109 |
| | Min | N.D | N.D | N.D | N.D | 0.018 | 0.073 | 0.117 | N.D | N.D | N.D | 0.080 | N.D | N.D | 0.008 | N.D | N.D | N.D |
| Tel Aviv | Mean | 0.332 | 0.707 | 0.076 | 2.080 | 0.497 | 0.256 | 0.861 | 0.035 | 0.008 | 0.007 | 0.524 | 0.002 | 0.008 | 0.032 | 0.004 | 0.003 | 0.010 |
| | Std | 0.374 | 0.767 | 0.052 | 1.261 | 0.626 | 0.163 | 0.705 | 0.034 | 0.005 | 0.008 | 0.356 | 0.002 | 0.011 | 0.017 | 0.004 | 0.005 | 0.012 |
| | Max | 1.822 | 3.593 | 0.198 | 5.720 | 2.585 | 0.775 | 4.006 | 0.175 | 0.023 | 0.026 | 1.719 | 0.007 | 0.043 | 0.089 | 0.020 | 0.017 | 0.058 |
| | Min | N.D | N.D | N.D | 0.309 | N.D | 0.033 | 0.064 | N.D | N.D | N.D | 0.026 | N.D | N.D | N.D | N.D | N.D | N.D |
| Haifa | Mean | 0.274 | 0.572 | 0.071 | 1.881 | 0.177 | 0.189 | 0.464 | 0.025 | 0.009 | 0.002 | 0.280 | 0.005 | 0.004 | 0.019 | 0.002 | 0.001 | 0.010 |
| | Std | 0.654 | 1.336 | 0.048 | 1.103 | 0.265 | 0.256 | 1.108 | 0.059 | 0.007 | 0.009 | 0.565 | 0.008 | 0.007 | 0.037 | 0.004 | 0.005 | 0.020 |
| | Max | 4.594 | 9.337 | 0.225 | 5.497 | 1.046 | 1.770 | 8.032 | 0.409 | 0.043 | 0.061 | 3.974 | 0.052 | 0.046 | 0.240 | 0.025 | 0.031 | 0.110 |

*not detected, min=minimum, Std=Standard deviation, Max=Maximum

Table S4. Inter-correlation among each pair of ion for each site

| Site | | | | $SO_4^{2-}$ | $NO_3^-$ | $Cl^-$ | $NH_4^+$ | $Na^+$ |
|---|---|---|---|---|---|---|---|---|
| Palestine | E. Jerusalem | $NO_3^-$ | Pearson Correlation | -0.06 | | | | |
| | | | p-value | 0.67 | | | | |
| | | $Cl^-$ | Pearson Correlation | -0.37 | 0.37 | | | |
| | | | p-value | 0.01 | 0.01 | | | |
| | | $NH_4^+$ | Pearson Correlation | 0.91 | -0.01 | -0.32 | | |
| | | | p-value | 0.00 | 0.92 | 0.02 | | |
| | | $Na^+$ | Pearson Correlation | 0.13 | 0.26 | 0.30 | -0.14 | |
| | | | p-value | 0.40 | 0.07 | 0.04 | 0.36 | |
| | | $K^+$ | Pearson Correlation | 0.19 | 0.29 | 0.14 | -0.03 | 0.30 |
| | | | p-value | 0.19 | 0.04 | 0.34 | 0.82 | 0.04 |
| | Hebron | $NO_3^-$ | Pearson Correlation | 0.21 | | | | |
| | | | p-value | 0.12 | | | | |
| | | $Cl^-$ | Pearson Correlation | -0.36 | 0.37 | | | |
| | | | p-value | 0.01 | 0.01 | | | |
| | | $NH_4^+$ | Pearson Correlation | 0.88 | 0.39 | -0.29 | | |
| | | | p-value | 0.00 | 0.00 | 0.04 | | |
| | | $Na^+$ | Pearson Correlation | -0.23 | 0.20 | 0.43 | -0.18 | |
| | | | p-value | 0.11 | 0.16 | 0.00 | 0.21 | |
| | | $K^+$ | Pearson Correlation | -0.09 | 0.06 | 0.07 | -0.13 | 0.39 |
| | | | p-value | 0.53 | 0.69 | 0.63 | 0.37 | 0.01 |
| | Noblus | $NO_3^-$ | Pearson Correlation | 0.08 | | | | |
| | | | p-value | 0.54 | | | | |
| | | $Cl^-$ | Pearson Correlation | -0.35 | 0.44 | | | |
| | | | p-value | 0.01 | 0.00 | | | |
| | | $NH_4^+$ | Pearson Correlation | 0.93 | 0.05 | -0.30 | | |
| | | | p-value | 0.00 | 0.71 | 0.02 | | |
| | | $Na^+$ | Pearson Correlation | 0.04 | 0.27 | 0.44 | -0.15 | |
| | | | p-value | 0.77 | 0.06 | 0.00 | 0.27 | |
| | | $K^+$ | Pearson Correlation | 0.39 | 0.39 | -0.06 | 0.17 | 0.31 |
| | | | p-value | 0.00 | 0.00 | 0.68 | 0.22 | 0.02 |

Table S4. Continued

| Site | | | | $SO_4^{2-}$ | $NO_3^-$ | $Cl^-$ | $NH_4^+$ | $Na^+$ |
|---|---|---|---|---|---|---|---|---|
| Jordan | Aqaba | $NO_3^-$ | Pearson Correlation | 0.32 | | | | |
| | | | p-value | 0.02 | | | | |
| | | $Cl^-$ | Pearson Correlation | -0.20 | 0.24 | | | |
| | | | p-value | 0.16 | 0.08 | | | |
| | | $NH_4^+$ | Pearson Correlation | 0.83 | 0.44 | -0.23 | | |
| | | | p-value | 0.00 | 0.00 | 0.11 | | |
| | | $Na^+$ | Pearson Correlation | -0.12 | 0.24 | 0.75 | -0.29 | |
| | | | p-value | 0.39 | 0.09 | 0.00 | 0.04 | |
| | | $K^+$ | Pearson Correlation | 0.41 | 0.55 | -0.06 | 0.32 | 0.00 |
| | | | p-value | 0.00 | 0.00 | 0.66 | 0.02 | 0.98 |
| | Rahma | $NO_3^-$ | Pearson Correlation | 0.16 | | | | |
| | | | p-value | 0.27 | | | | |
| | | $Cl^-$ | Pearson Correlation | -0.40 | 0.33 | | | |
| | | | p-value | 0.01 | 0.03 | | | |
| | | $NH_4^+$ | Pearson Correlation | 0.93 | 0.28 | -0.49 | | |
| | | | p-value | 0.00 | 0.04 | 0.00 | | |
| | | $Na^+$ | Pearson Correlation | -0.22 | 0.19 | 0.84 | -0.38 | |
| | | | p-value | 0.14 | 0.21 | 0.00 | 0.01 | |
| | | $K^+$ | Pearson Correlation | 0.27 | 0.47 | -0.02 | 0.30 | -0.10 |
| | | | p-value | 0.06 | 0.00 | 0.92 | 0.03 | 0.51 |
| | Amman | $NO_3^-$ | Pearson Correlation | 0.54 | | | | |
| | | | p-value | 0.00 | | | | |
| | | $Cl^-$ | Pearson Correlation | -0.03 | 0.47 | | | |
| | | | p-value | 0.80 | 0.00 | | | |
| | | $NH_4^+$ | Pearson Correlation | 0.88 | 0.65 | 0.06 | | |
| | | | p-value | 0.00 | 0.00 | 0.64 | | |
| | | $Na^+$ | Pearson Correlation | -0.11 | 0.15 | 0.51 | -0.26 | |
| | | | p-value | 0.46 | 0.31 | 0.00 | 0.06 | |
| | | $K^+$ | Pearson Correlation | 0.18 | 0.04 | 0.00 | -0.05 | 0.26 |
| | | | p-value | 0.19 | 0.78 | 0.98 | 0.70 | 0.07 |
| | Zarqa | $NO_3^-$ | Pearson Correlation | 0.35 | | | | |
| | | | p-value | 0.01 | | | | |
| | | $Cl^-$ | Pearson Correlation | 0.08 | 0.72 | | | |
| | | | p-value | 0.55 | 0.00 | | | |
| | | $NH_4^+$ | Pearson Correlation | 0.89 | 0.58 | 0.28 | | |
| | | | p-value | 0.00 | 0.00 | 0.04 | | |
| | | $Na^+$ | Pearson Correlation | -0.25 | 0.08 | 0.22 | -0.39 | |
| | | | p-value | 0.09 | 0.57 | 0.13 | 0.01 | |
| | | $K^+$ | Pearson Correlation | 0.06 | -0.17 | -0.09 | -0.03 | 0.09 |
| | | | p-value | 0.66 | 0.24 | 0.52 | 0.81 | 0.56 |

Table S4. Continued

| | Site | | | $SO_4^{2-}$ | $NO_3^-$ | $Cl^-$ | $NH_4^+$ | $Na^+$ |
|---|---|---|---|---|---|---|---|---|
| Israel | Eilat | $NO_3^-$ | Pearson Correlation | -0.34 | | | | |
| | | | p-value | 0.03 | | | | |
| | | $Cl^-$ | Pearson Correlation | -0.38 | 0.23 | | | |
| | | | p-value | 0.03 | 0.20 | | | |
| | | $NH_4^+$ | Pearson Correlation | 0.48 | 0.51 | -0.10 | | |
| | | | p-value | 0.00 | 0.00 | 0.57 | | |
| | | $Na^+$ | Pearson Correlation | -0.22 | 0.40 | 0.35 | -0.13 | |
| | | | p-value | 0.17 | 0.01 | 0.05 | 0.44 | |
| | | $K^+$ | Pearson Correlation | -0.07 | 0.58 | 0.14 | 0.44 | 0.10 |
| | | | p-value | 0.66 | 0.00 | 0.43 | 0.00 | 0.54 |
| | W. Jerusalem | $NO_3^-$ | Pearson Correlation | 0.20 | | | | |
| | | | p-value | 0.15 | | | | |
| | | $Cl^-$ | Pearson Correlation | -0.03 | 0.42 | | | |
| | | | p-value | 0.84 | 0.00 | | | |
| | | $NH_4^+$ | Pearson Correlation | 0.92 | 0.16 | -0.22 | | |
| | | | p-value | 0.00 | 0.25 | 0.12 | | |
| | | $Na^+$ | Pearson Correlation | 0.05 | 0.44 | 0.64 | -0.20 | |
| | | | p-value | 0.74 | 0.00 | 0.00 | 0.16 | |
| | | $K^+$ | Pearson Correlation | 0.18 | 0.35 | 0.65 | 0.00 | 0.40 |
| | | | p-value | 0.23 | 0.02 | 0.00 | 0.99 | 0.01 |
| | Tel Aviv | $NO_3^-$ | Pearson Correlation | 0.08 | | | | |
| | | | p-value | 0.55 | | | | |
| | | $Cl^-$ | Pearson Correlation | -0.19 | 0.41 | | | |
| | | | p-value | 0.18 | 0.00 | | | |
| | | $NH_4^+$ | Pearson Correlation | 0.90 | 0.35 | -0.21 | | |
| | | | p-value | 0.00 | 0.01 | 0.12 | | |
| | | $Na^+$ | Pearson Correlation | 0.10 | 0.42 | 0.91 | 0.03 | |
| | | | p-value | 0.45 | 0.00 | 0.00 | 0.83 | |
| | | $K^+$ | Pearson Correlation | 0.39 | 0.49 | 0.34 | 0.40 | 0.45 |
| | | | p-value | 0.00 | 0.00 | 0.01 | 0.00 | 0.00 |
| | Haifa | $NO_3^-$ | Pearson Correlation | 0.06 | | | | |
| | | | p-value | 0.67 | | | | |
| | | $Cl^-$ | Pearson Correlation | -0.27 | 0.17 | | | |
| | | | p-value | 0.07 | 0.26 | | | |
| | | $NH_4^+$ | Pearson Correlation | 0.93 | 0.09 | -0.44 | | |
| | | | p-value | 0.00 | 0.51 | 0.00 | | |
| | | $Na^+$ | Pearson Correlation | 0.20 | 0.36 | 0.63 | 0.13 | |
| | | | p-value | 0.15 | 0.01 | 0.00 | 0.35 | |
| | | $K^+$ | Pearson Correlation | 0.52 | 0.42 | 0.09 | 0.52 | 0.44 |
| | | | p-value | 0.00 | 0.00 | 0.58 | 0.00 | 0.00 |

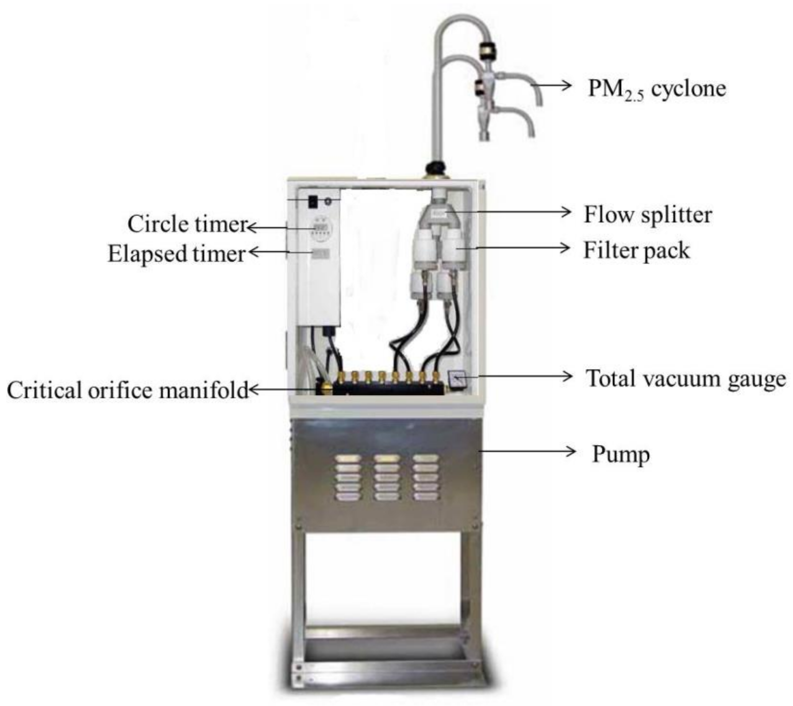


**Fig. S1**. $PM_{2.5}$ sampling system modified from URG-3000ABC (Chapel Hill, NC, USA; www.urgcorp.com).

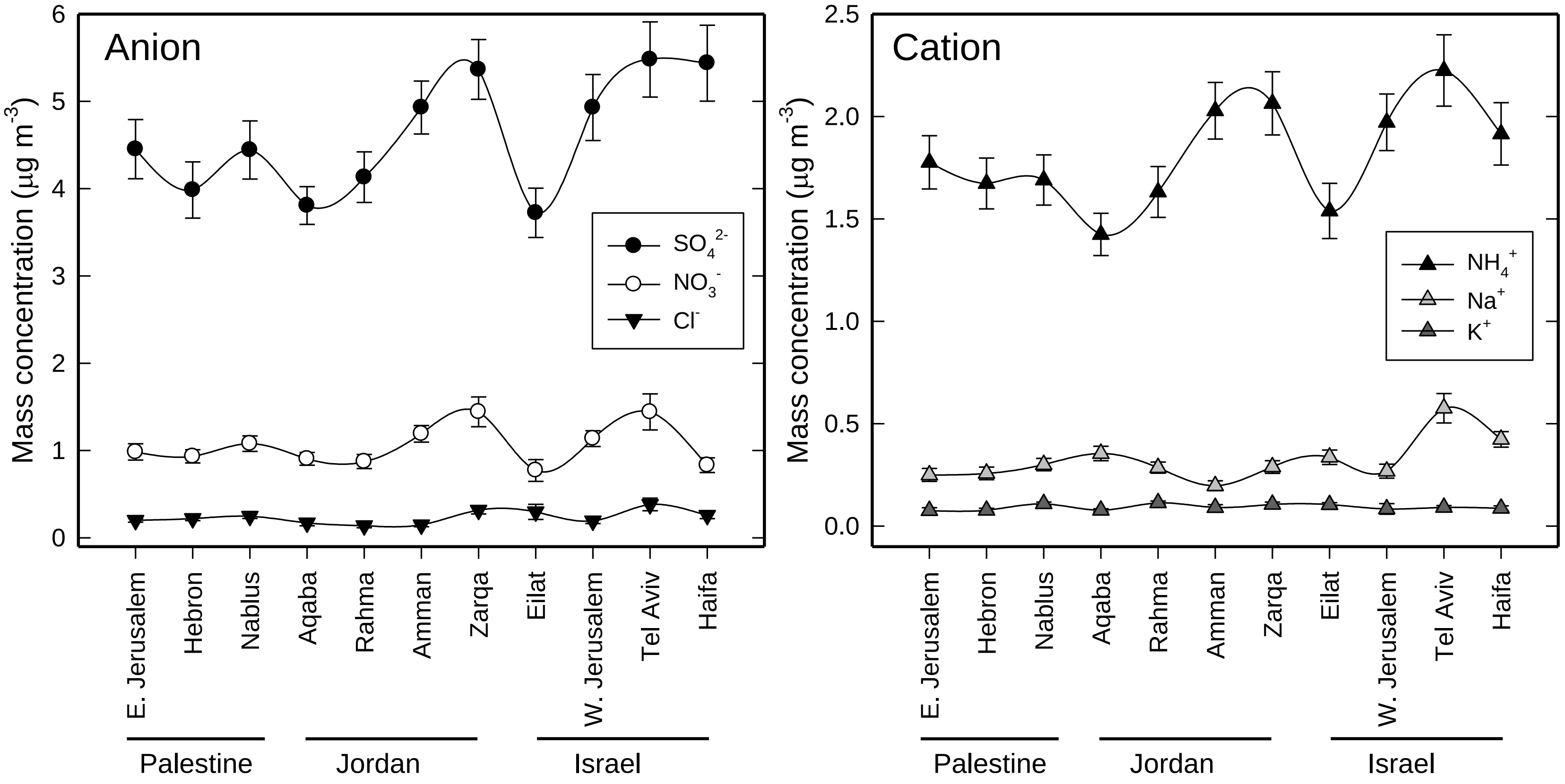


**Fig. S2**. Annual trends for inorganic water soluble ions at each site.

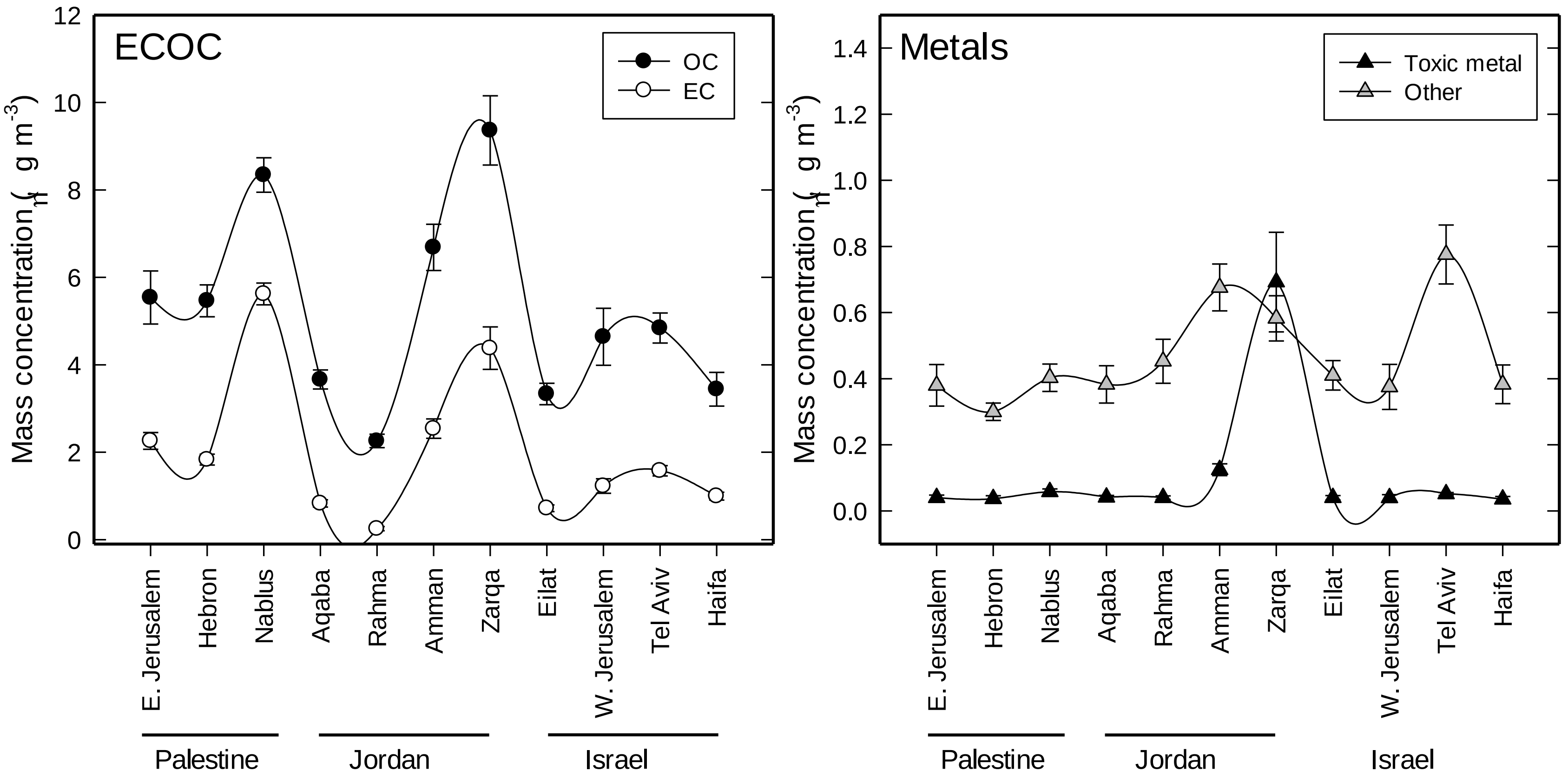


**Fig. S3**. Annual trends for ECOC and metals at each site.

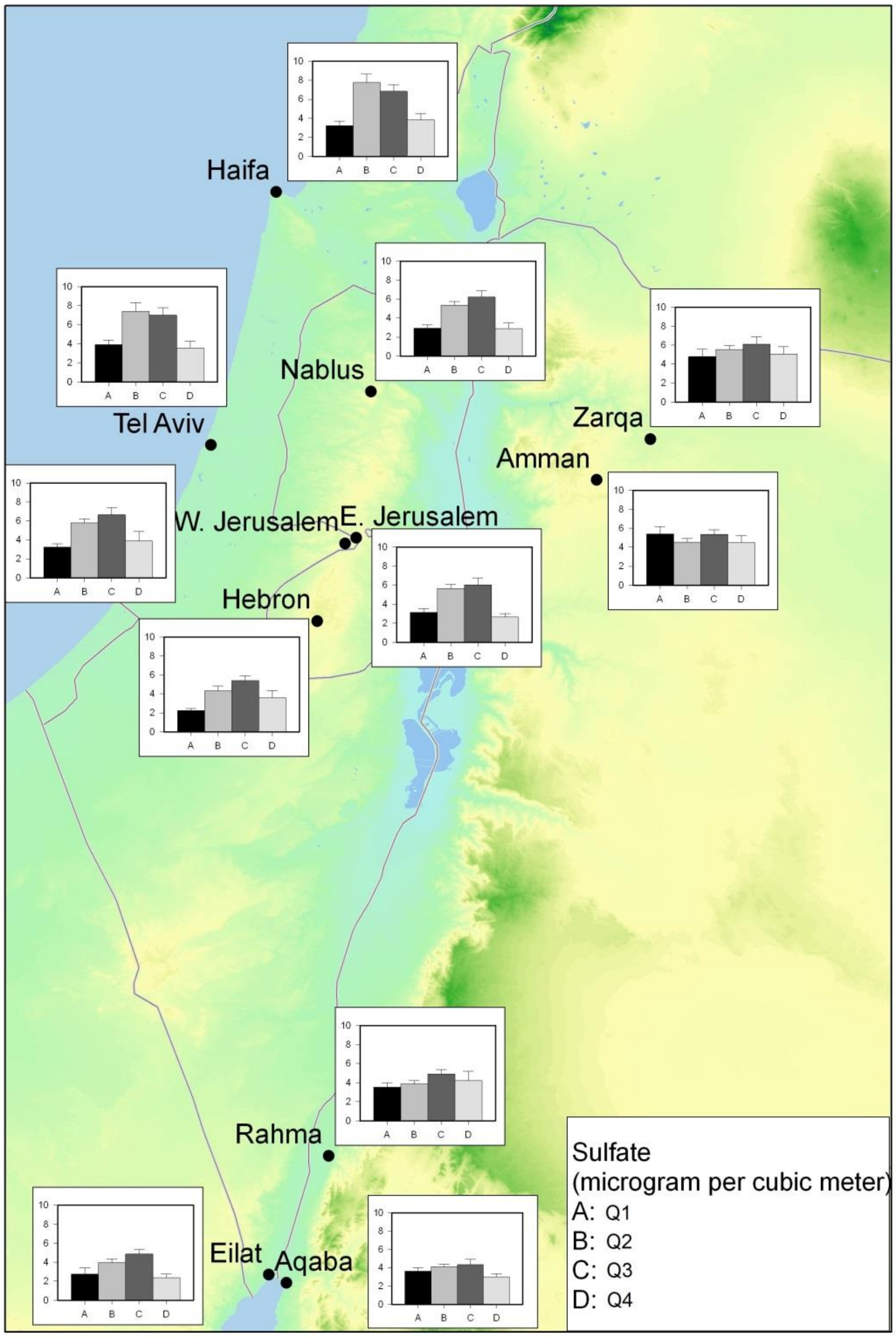


**Fig. S4**. Spatial and seasonal trends of $PM_{2.5}$ sulfate concentrations at each site.

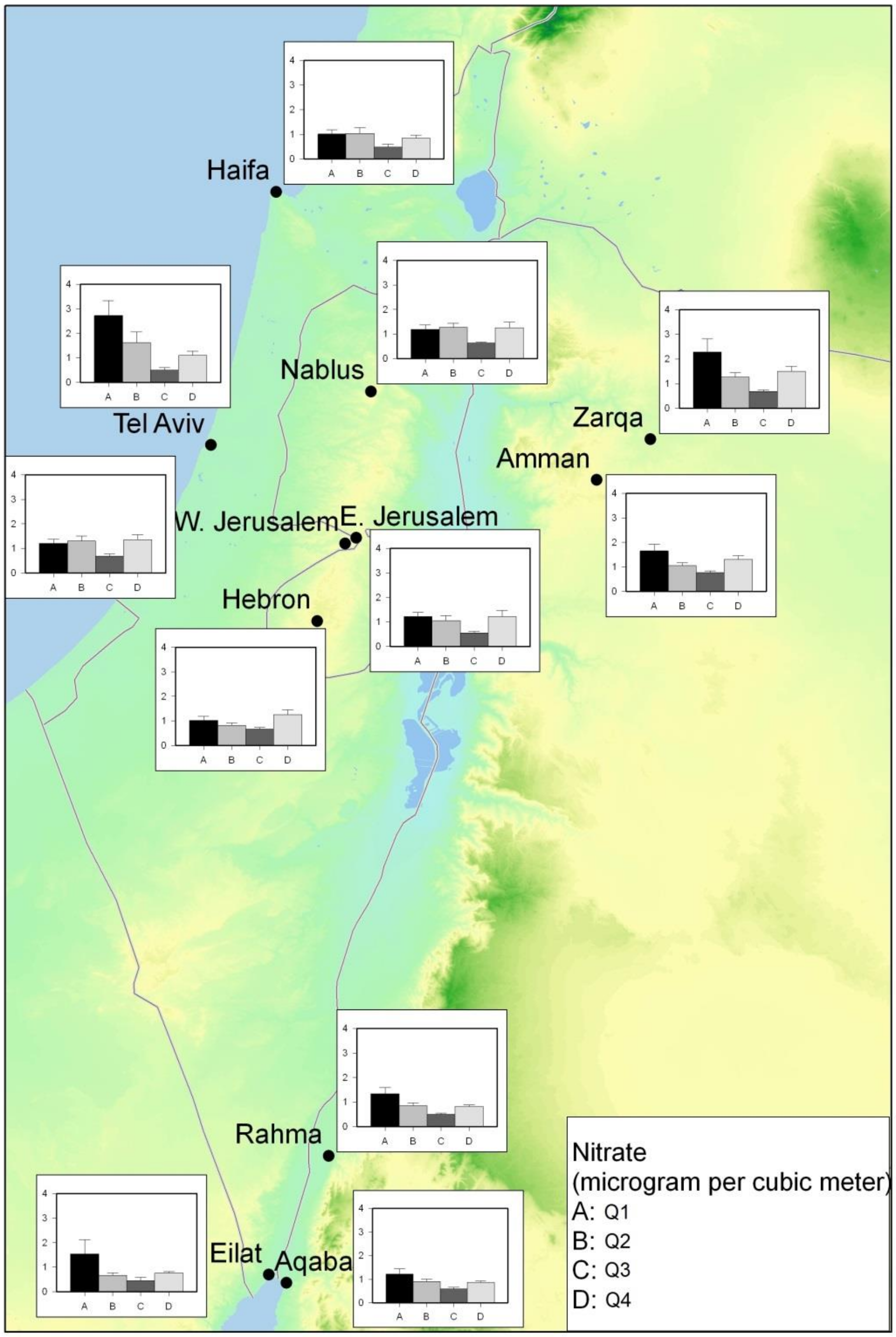


**Fig. S5**. Spatial and seasonal trends of $PM_{2.5}$ nitrate concentrations at each site.

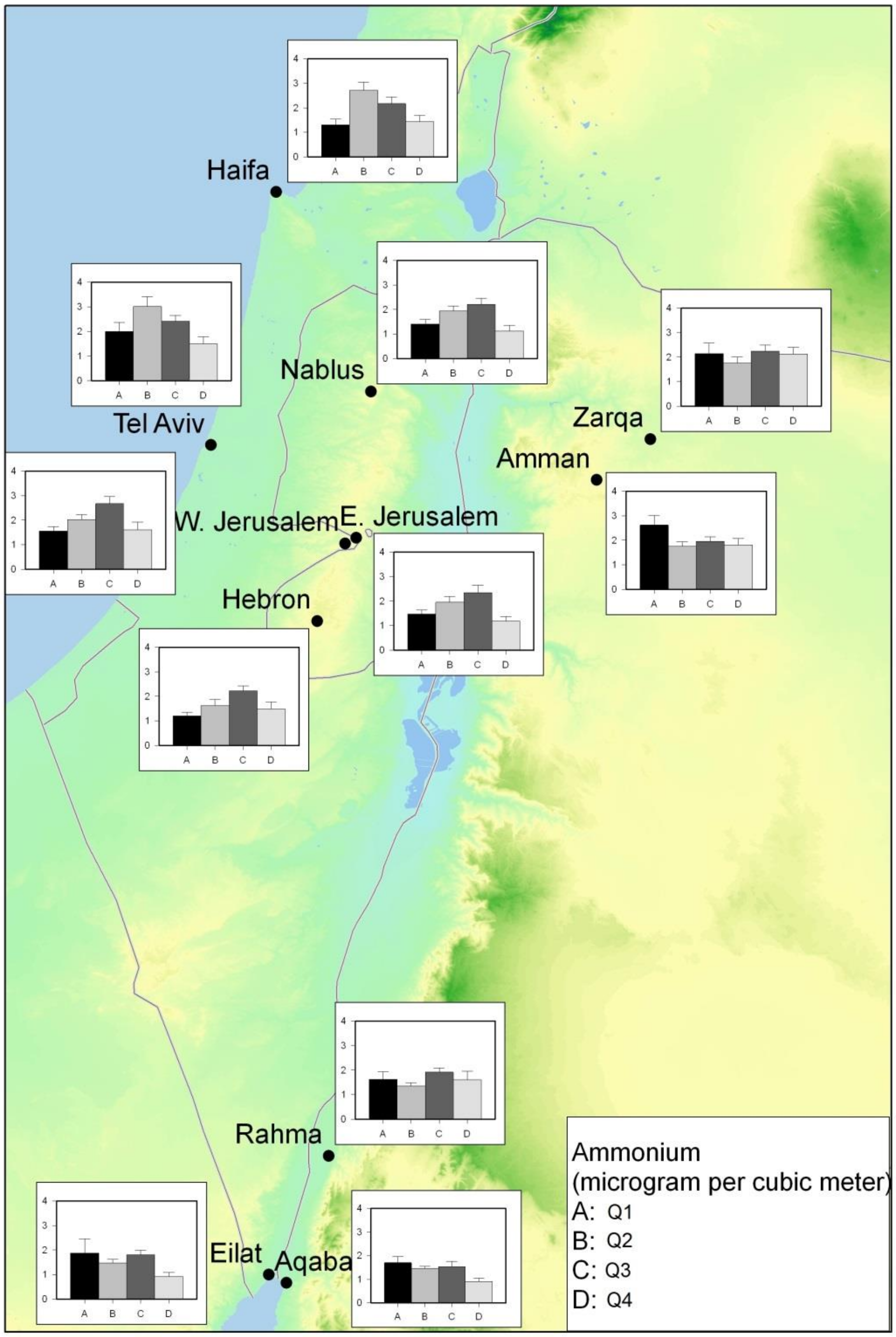


**Fig. S6**. Spatial and seasonal trends of $PM_{2.5}$ ammonium concentrations at each site.
